# Factors associated with cesarean delivery during labor in primiparous women assisted in the Brazilian Public Health System: data from a National Survey

**DOI:** 10.1186/s12978-016-0231-z

**Published:** 2016-10-17

**Authors:** Marcos Augusto Bastos Dias, Rosa Maria Soares Madeira Domingues, Arthur Orlando Corrêa Schilithz, Marcos Nakamura-Pereira, Maria do Carmo Leal

**Affiliations:** 1 Instituto Fernandes Figueira/FIOCRUZ, Av. Rui Barbosa 716, Rio de Janeiro, CEP: 22250-020 Brasil; 2 Instituto Nacional de Infectologia Evandro Chagas/FIOCRUZ, Av. Brasil, 4365 - Manguinhos, Rio de Janeiro, CEP: 21040-360 Brasil; 3 Escola Nacional Saúde Publica Sérgio Arouca/FIOCRUZ, Av. Brasil, 4365 - Manguinhos, Rio de Janeiro, CEP: 21040-360 Brasil

**Keywords:** Cesarean delivery, Primiparous, Labour assistance, Nurse midwife, Good practices

## Abstract

**Background:**

The rate of cesarean delivery (CD) in Brazil has increased over the past 40 years. The CD rate in public services is three times above the World Health Organization recommended values. Among strategies to reduce CD, the most important is reduction of primary cesarean. This study aimed to describe factors associated with CD during labor in primiparous women with a single cephalic pregnancy assisted in the Brazilian Public Health System (SUS).

**Methods:**

This study is part of the Birth in Brazil survey, a national hospital-based study of 23,894 postpartum women and their newborns. The rate of CD in primiparous women was estimated. Univariate and multivariable logistic regression was performed to analyze factors associated with CD during labor in primiparous women with a single cephalic pregnancy, including estimation of crude and adjusted odds ratios and their respective 95 % confidence intervals.

**Results:**

The analyzed data are related to the 2814 eligible primiparous women who had vaginal birth or CD during labor in SUS hospitals. In adjusted analyses, residing in the Southeast region was associated with lower CD during labor. Occurrence of clinical and obstetric conditions potentially related to obstetric emergencies before delivery, early admission with < 4 cm of dilatation, a decision late in pregnancy for CD, and the use of analgesia were associated with a greater risk for CD. Favorable advice for vaginal birth during antenatal care, induction of labor, and the use of any good practices during labor were protective factors for CD. The type of professional who attended birth was not significant in the final analyses, but bivariate analysis showed a higher use of good practices and a smaller proportion of epidural analgesia in women cared for by at least one nurse midwife.

**Conclusions:**

The CD rate in primiparous women in SUS in Brazil is extremely high and can compromise the health of these women and their newborns. Information and support for vaginal birth during antenatal care, avoiding early admission, and promoting the use of good practices during labor assistance can reduce unnecessary CD. Considering the experience of other countries, incorporation of nurse midwives in childbirth care may increase the use of good practices during labor.

**Electronic supplementary material:**

The online version of this article (doi:10.1186/s12978-016-0231-z) contains supplementary material, which is available to authorized users.

## Background

In modern obstetrics, cesarean delivery (CD) as a safe form of birth is responsible for reducing maternal and neonatal mortality [1]. However, Ye et al. [2] have shown that increasing the CD rate above 10 % shows no benefit to the woman or the newborn, and may be associated with maternal and neonatal complications and increased health system costs [3]. Unnecessary CD may also compromise the reproductive future of women [4].

The CD rate in Brazil has increased in a steadily manner over the previous past 40 years, and from 38 % in 2000 to 50.6 % in 2009 when this surgery became the main mode of delivery in the country [5]. In 2013, 56.6 % of births were CD [6], and a study conducted by the World Health Organization (WHO) estimated that in Brazil in 2008 there were approximately 1,000,000 unnecessary CDs [7].

In Brazil, although the CD rate in private hospitals is the highest, in public services, it is approximately three times above the recommended rate [4]. The Brazilian Public Health System (SUS) is publicly funded, free of any cost and had a CD rate of 37,1 % in 2010. The Private Health System participate complementary in the health assistance, is responsible for almost 20 % of all births and had a CD rate of 63,6 % in the same year [5].

The obstetric model of care delivery has been identified as one of the causes for the increase in CD rate [8–10]. In Brazilian hospitals, the use of good practices in delivery care is still quite low, despite evidence that these practices are associated with lower intervention rates, higher maternal satisfaction, and good perinatal outcomes [11].

In primiparous, whose labor and delivery tend to be longer, the occurrence of CD and other maternal and perinatal outcomes is directly related to the quality of care. A study by Leal et al. [11] showed that in Brazil, primiparous women were admitted earlier and were more exposed to hospital intervention during labor and birth compared with multiparous women. Different authors have highlighted the key role of reducing primary cesarean to reduce CD rates [12].

The objective of this study was to estimate the rate of elective and intrapartum CD in primiparous women with a singleton pregnancy and a fetus in the vertex presentation assisted in the Brazilian Public Health System (SUS) and describe the factors associated with CD during labor and childbirth care.

## Methods

This study is part of the Birth in Brazil survey, a national hospital-based study of a sample of postpartum women and their newborns that was conducted between February 2011 and October 2012. Hospitals with 500 or more annual deliveries were selected and stratified according to region, location (state capital or non-capital city), and type of healthcare (private, public, and mixed). A total of 23,894 postpartum women were interviewed in 266 hospitals that were distributed in all Brazilian states. Further details regarding the sample design have been reported by Vasconcellos et al. [13]. In the first phase of the study, face-to-face interviews were conducted with the postpartum women during their hospital stay and data were collected from the mother’s and newborn’s medical records. Hand-held maternity notes were photographed. Further details on data collection were provided by do Carmo Leal et al. [14].

**Fig. 1:**
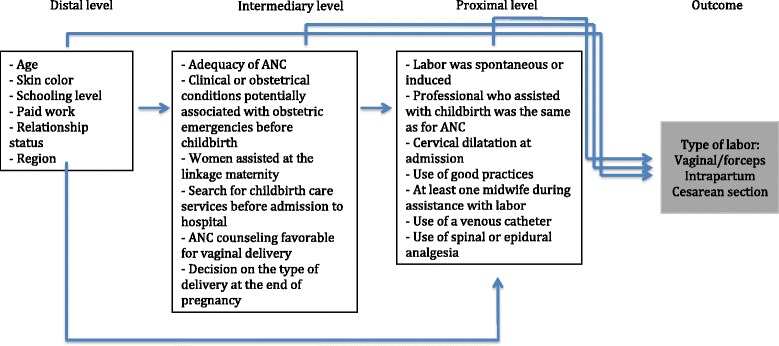
Hierarchical model of analyses of primiparous women with a single cephalic pregnancy

Data from primiparous women who had a single birth with the fetus in cephalic presentation born alive, or as a stillbirth after 22 weeks of pregnancy and assisted in the SUS were analyzed. Women who self-reported as “East Asian” or indigenous were excluded because of the small proportion of these participants in the sample (1.1 and 0.4 % of the sample total, respectively). Univariate and unconditional multivariable logistic regression analysis of factors associated with intrapartum CD in primiparous women who were assisted in the SUS were made using a theoretical model with three levels of hierarchy [15] (Fig. 1). On the distal level, maternal sociodemographic variables were included as follows: “maternal age” (12–19 years, 20–34 years, or ≥35 years); “schooling level” (up to 7 years, 8–10 years, 11–14 years, and ≥15 years of school attendance); “self-reported skin color” (white, black, or brown); “paid work” (yes or no); “relationship status” (living or not with partner); and “macro region of Brazil” (North, South, Northeast, Southeast, and Midwest). On an intermediate level, the characteristics of pregnancy, use of antenatal care (ANC), and childbirth care services-related variables were included as follows: “adequacy of ANC” (considering a minimum of seven consultations and routine exams for a term pregnancy, adjusted for gestational age at birth assessed according to national protocols [16]); “clinical or obstetric conditions potentially associated with obstetric emergencies before childbirth” (placenta previa, placental abruption, hypertensive syndromes, diabetes, maternal infection, and HIV infection); “women assisted at the linkage maternity” (if the woman was assisted at reference maternity for childbirth care as guidance provided by ANC, yes or no); “search for childbirth care services before hospital admission” (search of more than one maternity service during labor for childbirth care, yes or no); “ANC counseling favorable for vaginal delivery” (yes or no); and “decision on the type of delivery at the end of pregnancy” (if at the end of pregnancy during antenatal care the woman had already decided for a type of birth; no, yes vaginal birth, yes cesarean delivery). On the proximal level, we included variables that were associated with labor and the mode of birth: “spontaneous or induced labor”; “the same professional provided assistance during ANC and childbirth care” (yes or no); “cervical dilatation at admission” (<4 cm—early admission or ≥ than 4 cm); “use of any of the good practices during labor” (eating, mobility, use of nonpharmacological pain relief, presence of companionship, and monitoring progress of labor using a partogram); “presence of at least one nurse midwife during assistance with labor “(yes or no); “use of a venous catheter” (yes or no); and “use of spinal or epidural analgesia” (yes or no). The outcome was the mode of delivery (vaginal birth or CD during labor). CDs were classified based on information recorded in hospital files. CDs were defined as intrapartum if the woman had a spontaneous or induced labor and underwent cesarean section when uterine dilatation was at least 4 cm [17]. All other CDs were classified elective, no matter the duration of labor or the indication of the CD. The variables “clinical or obstetrical conditions potentially associated with obstetric emergencies before childbirth”, “spontaneous or induced labor”, “cervical dilatation at admission”, “monitoring progress of labor using a partogram”, “presence of at least one midwife during assistance with labor “,“use of a venous catheter”, “use of spinal or epidural analgesia” and “type of birth” were based on information recorded in hospital files. All other variables were self-reported by woman during the interview. Forceps deliveries were analyzed together with other vaginal births because of their small number (1 %). There were no vacuum deliveries.

Univariate analysis was used to estimate the unadjusted odds ratio (OR) and 95 % confidence interval (95 % CI). In the first multivariable model, all distal variables were included. Variables of the distal level with a significance level estimated as lower than 0.20 were included in the second model, which also included all of the variables of the intermediate level. In the third model, distal and intermediate variables with a significance level estimated as lower than 0.20 and the proximal variables were included. All variables with a significance level of <0.05 and some variables of interest according to the theoretical model were retained in the final multivariable model. The results from the final multivariable model are expressed as adjusted ORs with their corresponding 95 % CIs.

To analyze the association of the use of good practices during labor and the use of spinal or epidural analgesia with the professional who assisted labor, the chi-square test was used to determine differences between proportions with a significance level of <0.05.

In all of the statistical analyses, the complex sampling design was taken into consideration. Data weighting was calculated according to the inverse of the probability of inclusion of each puerperal woman in the sample. To ensure that the distribution of puerperal women who were interviewed was similar to that observed among the births in the population sampled in 2011, a calibration procedure was used in each selection stratum [13]. The weighting and calibration procedure have been used for all data (numbers and percentage) presented in this study.

Analyses were performed using IBM SPSS software version 20.0 (IBM Corp., Armonk, USA).

This study was approved by the Research Ethics Committee of ENSP/Fiocruz, under report no. 92/2010. Care was taken to ensure privacy and confidentiality regarding the information collected from the women. Informed consent was obtained before the interview through the use of a informed consent statement.

## Results

**Fig. 2:**
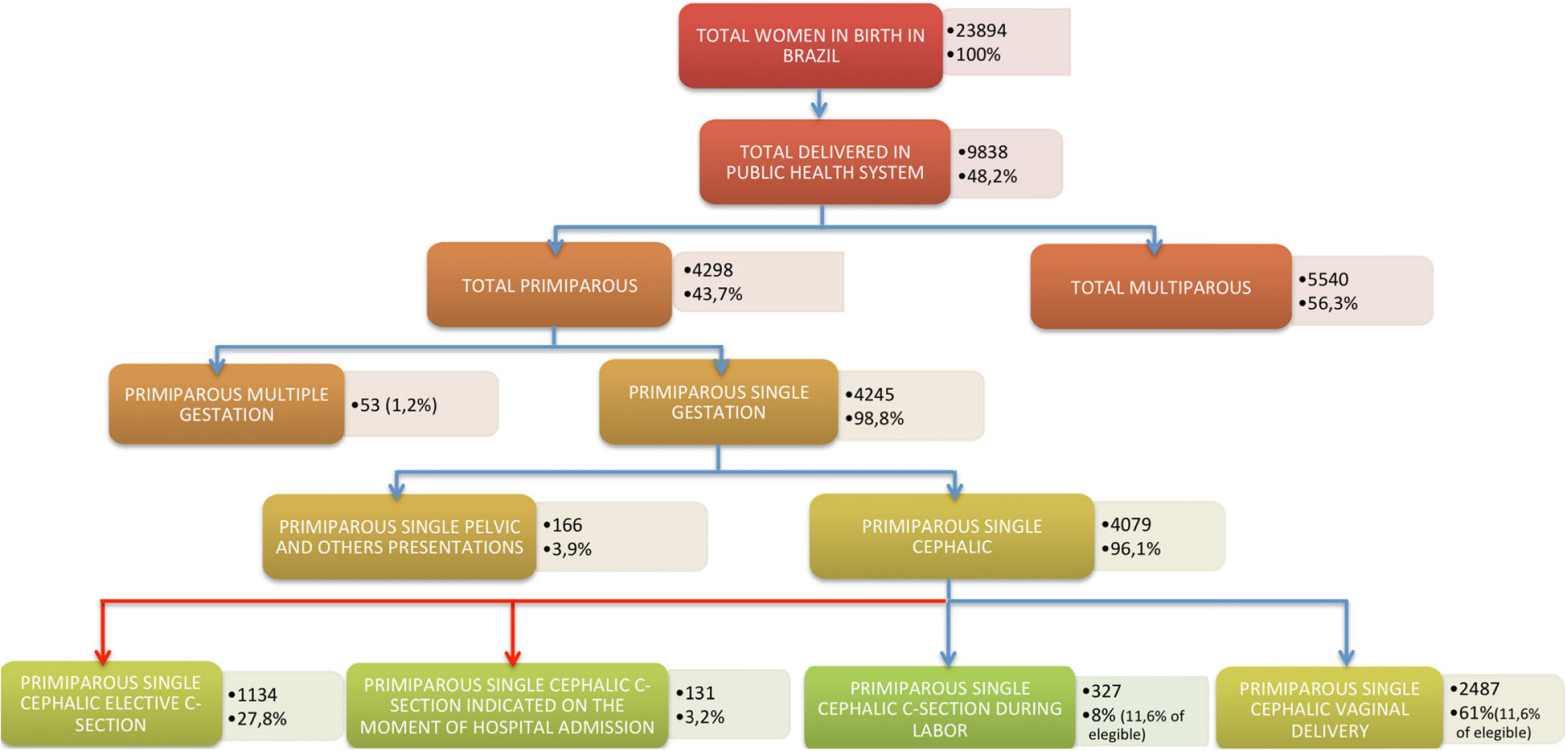
Distribution of women in the study sample according to place of delivery, parity and other obstetric categories

From the 23,894 women interviewed in the Birth in Brazil survey, 9838 (41.2 %) were attended in SUS hospitals. From a total of 4298 primiparous women who delivered in SUS hospitals 4079 (96,1 %) had a single pregnancy with cephalic presentation. Of these, 1592 (39 %) underwent CD, most of the surgeries (1134, 27.8 %) occurred before onset of labor and 131 (3.2 %) had an indication for CD at the moment of admission to hospital. The number of primiparous women with a single cephalic gestation who had an indication for CD during labor was 327 (11,6 % of eligible women). The analyzed data are related to the 2814 primiparous women with a singleton pregnancy and a fetus in cephalic presentation who had vaginal birth or CD during labor in SUS hospitals. Figure 2 summarizes the distribution of pregnant women who were assisted in SUS hospitals.

Almost half (47,7 %) of the respondents were up to 19 years old, 51 % were between 20 and 34 years and only 1 % were 35 years or older. The majority (65.2 %) of women self-reported their skin color as brown, had up to 8 years of education (58.1 %), lived with a partner (71.9 %), and had no paid work (73.7 %). Most primiparous women lived in the Northeast region (37.7 %) followed by the Southeast (36.6 %), the North (12.7 %), the South (6.7 %), and the Midwest (6.3 %).

Almost all (99 %) primiparous women had had ANC, while only 7.2 % had their ANC considered appropriate when assessed according to the criteria proposed in the national protocol [15]. Most (77 %) primiparous reported that they were assisted at the linkage maternity and 19.4 % had to search for childbirth care services before admission to hospital. Clinical and obstetric conditions potentially related to obstetric emergencies before delivery were present in 13.2 % of primiparous women and 4.5 % of them had already opted for CD in late pregnancy. Counseling was favorable for vaginal birth during ANC to 72,1 % of primiparous women and 61.2 % had opted for a vaginal birth in late pregnancy. Approximately 17 % of primiparous women had their labor induced, 1.9 % were cared for by the same professional during ANC and childbirth, and 37.6 % had early admission to the maternity with < 4 cm of cervical dilatation. Whilst being cared for during labor, 85 % of the primiparous women had access to at least one good practice (30.4 % had received fluids or food, 48 % were able to ambulate, 36.4 % used some non-pharmacological pain relief, in 52.6 %, a partogram was used to monitor labor, and 56.4 % had companionship of her choice). A venous catheter was sited in 67.9 % of primiparous women, 9.3 % made use of analgesia, and 18.7 % had assistance with at least one midwife whilst being cared for labor.

Among all of the distal factors analyzed, only the mother’s region of residence showed a significant difference according to the main outcome of CD during labor.

Primiparous women living in the Southeast region had a rate of 6.7 % for CD, followed by residents of the Northeast with a rate of 13.9 %, 14 % in the Northern region, 15.3 % in the Southern region, and the highest rate of 18.6 % in the Midwest region (Table 1).

**Table 1:** Maternal variables of primiparous women assisted during labor in public hospitals, Brazil, 2011–2012 (*N* = 2487)

		Mode of delivery				
		Cesarean during labor		Vaginal delivery		*p* value *
		N	%	N	%	
Sociodemographic variables						
Maternal age (years)	12–19	144	10.7	1198	89.3	0.372
	20–34	180	12.5	1264	87.5	
	≥35	3	10.7	25	89.3	
Self-reported skin color	White	69	10.4	595	89.6	0.415
	Black	27	9.2	267	90.8	
	Brown	223	12.4	1569	87.6	
Schooling level	0–7 years	81	11.1	650	88.9	0.753
	8–10 years	98	10.9	803	89.1	
	11–14 years	141	12.5	984	87.5	
	15 or more	6	11.5	46	88.5	
Paid Work	Yes	89	12.1	649	87.9	0.728
	No	237	11.4	1836	88.6	
Relationship status	Not living with partner	83	10.5	708	89.5	0.421
	Living with partner	243	12.0	1777	88.0	
Macro region of Brazil	North	50	14.0	308	86.0	0.037
	Northeast	147	13.9	913	86.1	
	South	29	15.3	161	84.7	
	Southeast	69	6.7	960	93.3	
	Midwest	33	18.6	144	81.4	
Pregnancy characteristics, use of ANC, and childbirth care services						
Adequacy of ANC	Inadequate	307	11.8	2305	88.2	0.590
	Adequate	20	9.9	182	90.1	
Clinical or obstetrical conditions potentially associated with obstetric emergencies before childbirth	Yes	85	22.8	288	77.2	<0.001
	No	242	9.9	2199	90.1	
Women assisted at the linkage maternity	Yes	141	12.6	980	87.4	0.893
	No	41	12.2	294	87.8	
Search for childbirth care services before hospital admission	Yes	76	13.9	470	86.1	0.134
	No	250	11.0	2016	89.0	
Prenatal counseling favorable for vaginal delivery	Yes	175	8.6	1854	91.4	<0.001
	No	152	19.4	633	80.6	
Decision on the type of delivery at the end of pregnancy	No	167	17.5	790	82.5	<0.001
	Yes, vaginal	100	5.9	1608	94.1	
	Yes, cesarean	58	45.7	69	54.3	
Labor and mode of delivery variables						
Type of labor	Spontaneous	306	13.2	2018	86.8	<0.001
	Induced	21	4.3	468	95.7	
Same professional provided assistance during ANC and childbirth	Yes	12	22.2	42	77.8	0.152
	No	313	11.4	2442	88.6	
Cervical dilation at admission	≥4 cm	171	9.7	1584	90.3	0.017
	<4 cm	156	14.7	902	85.3	
Use of any of the good practices during labor	Yes	207	8.7	2185	91.3	<0.001
	No	120	28.5	301	71.5	
Use of a venous catheter	Yes	233	12.3	1656	87.7	0.507
	No	94	10.5	800	89.5	
Use of spinal or epidural analgesia	Yes	79	30.4	181	69.6	<0.001
	No	249	9.9	2276	90.1	
Presence of at least one obstetric nurse during assistance with labor	Yes	30	6.4	442	93.6	0.003
	No	276	13.5	1774	86.5	
Total		327	11.6	2487	88.4	

* Chi-square of Pearson was used for analysis

In the intermediate level, among the characteristics related to current pregnancy, the clinical and obstetric conditions potentially related to obstetric emergencies and the decision by a CD at the end of gestation showed associations with a higher occurrence of CD during labor. Favorable advice for vaginal birth was protective for the occurrence. Adequacy of ANC, have being assisted at the linkage maternity, and a search for more than one service for care during labor and birth showed no significant association with CD during labor (Table 1).

Among factors related to assistance with labor (Table 1), having induction of labor (IOL), using at least one good practices during labor, and the presence of at least one nurse midwife during labor were protective factors for CD. Early hospital admission, with cervical dilation < 4 cm, and the use of analgesia during labor were associated with a higher occurrence of CD during labor. Having had the same professional for ANC assistance and delivery and use of a venous catheter showed no significant association with the outcome.

The results of the multivariable logistic regression (Table 2) showed that among distal level variables, only the region of residence remained significant for an increased risk for CD during labor when comparing the Southeast region with the Northeast (OR 2.3; 95 % CI 1.1–4.7), North (OR 2.5; 95 % CI 1.3–4.6), South (OR 2.8; 95 % CI 1.4–5.5), and Midwest regions (OR 3.4; 95 % CI 1.6–7.1). Occurrence of clinical and obstetric conditions potentially related to obstetric emergencies before delivery (OR 2.7; 95 % CI 1.7–4.4), early admission with < 4 cm of dilatation (OR 2.1; 95 % CI 1.4–3.3), the decision in late pregnancy for a CD (OR 12.1; 95 % CI 7.1–20.7), and the use of analgesia (OR 3.7; 95 % CI 1.8–7.6) were associated with a greater risk for a CD. Favorable advice for vaginal delivery during ANC (OR 0.5; 95 % CI 0.3–0.6), IOL (OR 0.2; 95 % CI 0.1–0.4), and the use of at least one good practice during labor (OR 0.3; 95 % CI 0.2–0.5) were protective factors for CD.

**Table 2:** Multiple logistic regression of primiparous assisted during labor in public hospitals, Brazil, 2011–2012 (*N* = 2487)

			Mode of delivery		
		Crude OR ^b^	Adjusted OR	95 % CI	
				Lower	Upper
Distal factors					
Macro region of Brazil	North	2,2	2.5	1.3	4.6
	Northeast	2,2	2.3	1.1	4.7
	South	2,5	2.8	1.4	5.5
	Southeast ^a^	1	1	-	-
	Midwest	3,2	3.4	1.6	7.1
Intermediate factors					
Clinical or obstetrical conditions potentially associated with obstetric emergencies before childbirth	Yes	2,7	2.7	1.7	4.4
	No ^a^	1	1	-	-
Prenatal counseling favorable for vaginal delivery	Yes	0,4	0,5	0.3	0.6
	No ^a^	1	1	-	-
Decision on the type of delivery at the end of pregnancy	No	3,4	3.5	2.6	4.5
	Yes, vaginal ^a^	1	1	-	-
	Yes, cesarean	13,6	12.1	7.1	20.7
Proximal factors					
Type of labor	Spontaneous ^a^	1	1	-	-
	Induced	0,3	0,2	0,1	0,4
Cervical dilation at admission	≥4 cm ^a^	1	1	-	-
	<4 cm	1,6	2.1	1.4	3.3
Use of any of the good practices during labor	Yes	0,2	0,3	0,2	0,5
	No ^a^	1	1	-	-
Use of spinal or epidural analgesia	Yes	4,0	3.7	1.8	7.6
	No ^a^	1	1	-	-
Presence of at least one midwife during assistance with labor	No	2,3	1.0	0.6	1.7
	Yes ^a^	1	1	-	-

^a^ Reference category

^b^ All significative coeficients at 0,05

The type of professional who attended birth was not significant in the final analyses (OR 1.0; 95 % CI 0.6–1.7), but we decided to keep this variable in the final model because of its relevance. In the bivariate analysis, women who had the presence of at least one midwife during labor had increased use of good practices and a smaller proportion of epidural analgesia (Table 3). A higher proportion of midwives caring for women during labor in the Southeast, the country region with the lowest CD rate, was as also observed (36,9 % data not shown in tables).

**Table 3:** Bivariate analyses of primiparous assisted during labor in public hospitals, Brazil, 2011–2012 (*N* = 2487)

		Professional who assisted with labor				
		Physician		Midwife		*p* value*
		N	%	N	%	
Use of any of the good practices during labor	Yes	1709	79.4	443	20.6	0.001
	No	342	92.2	29	7.8	
Eating	Yes	541	71.4	217	28.6	0.002
	No	1509	85.6	254	14.4	
Mobility	Yes	961	80.2	238	19.8	0.418
	No	1089	82.3	234	17.7	
Use of nonpharmacological pain relief	Yes	702	74.3	243	25.7	0.006
	No	1349	85.5	229	14.5	
Presence of companionship	Yes	1101	82.8	229	17.2	0.327
	No	790	77.7	227	22.3	
Monitoring progress of labor using a partogram	Yes	1048	74.7	355	25.3	<0.001
	No	1003	89.6	117	10.4	
Use of spinal or epidural analgesia	Yes	232	92.8	18	7.2	0.002
	No	1818	80.1	453	19.9	
Total		2050	81.3	472	18.7	

*Chi-square of Pearson was used for analysis

## Discussion

This study estimated the rate of elective (27.8 %) and intrapartum CD (8 %) in primiparous women with a singleton pregnancy and a fetus in the vertex presentation assisted in the public health services in Brazil. The total rate of 35.8 % is high when compared to studies with similar populations. Bryant et al. [18] found a CD rate of 17.8 % in low risk primiparous in a retrospective cohort study of women delivering at the University of California, San Francisco, between 1980 and 2001 and O’Neill et al [19] identified a rate of 12.50 % emergency CD and 4.68 % elective, between 1982 and 2010, in a cohort of all live births in primiparous women in Denmark.

Most of the CD in our study was elective but in this analysis, we have identified the factors associated with intrapartum CD. The occurrence of clinical and obstetric conditions potentially related to obstetric emergencies before delivery, a decision in late pregnancy for CD, early admission with < 4 cm of dilatation, and the use of analgesia were associated with a greater proportion of CD. Residing in southeast region, favorable advice for vaginal birth during antenatal care, induction of labor, and the use of any of the good practices during labor were protective factors for CD.

Clinical and obstetric conditions are known risk factors for CD, both elective and intrapartum [18, 19]. We didn’t analyze the CD indications and the management of the clinical and obstetric conditions during antenatal and labor care. Brazil has a high coverture of ANC and in our study, almost all primiparous women had received at least one ANC consultation. However, less than 10 % had an adequate or more than adequate ANC when assessed according to national protocols. Other authors [20, 21] have also pointed out the high rate of inadequacy of ANC in Brazil. This inadequacy can result in adverse outcomes, as many routine practices during ANC are associated with lower rates of maternal mortality and fetal losses [22]. In our study, ANC was not associated with intrapartum CD. This may be the result of residual confounding, as the adequacy score used did not assess the management adequacy of specific conditions.

Antenatal care is not only important for the adequate care of obstetric and clinical complications. Our results show that the receipt of information supportive of a vaginal birth and the decision to choose a vaginal birth at the end of pregnancy were associated with a lower rate of intrapartum CD. Domingues et al. [23] found that women in the public sector in Brazil are not supported in their choice of vaginal birth, because their preferences for this type of birth diminish during pregnancy. Fear of pain in childbirth is the main reason for women preferring a CD in Brazil and the lack of support during pregnancy may discourage woman, as the birth gets closer.

Induction of labor (IOL) was associated with a lower rate of intrapartum CD. This is a common procedure in different countries and can modify the maternal and perinatal outcomes [24]. The rate of IOL in our study was 17.4 % and is lower than that found by other authors in similar groups: greater than 23 % for all pregnancies with single fetuses in the USA in 2010 [25]; 29 % in women with 32 or more weeks of gestation in 2007 in Australia [24]. One possible explanation for this smaller induction rate was the high rate of elective CD found in our study. In studies with high- and low-risk pregnancies [26] or term and post-term pregnancies [27], IOL was associated with a lower CD rate than expectant management.

Early admission in labor with < 4 cm of dilation is a known risk for CD [28] and occurred in 37.6 % of women in our sample. Women who are admitted to hospital in the latent phase (<3 cm cervical dilation) have a higher risk of obstetrical interventions, including electronic fetal monitoring, epidural analgesia, oxytocin and CD, than those who are admitted in active labor [29]. Neal et al. [30] suggested an evidence-based standardized approach for admission for labor to decrease inadvertent admissions of women in pre-active labor: when one cannot diagnose active labor with relative certainty, observation before admission is warranted.

The use of any of the good practices during labor was associated with lower rates of CD. In the Birth in Brazil study, Leal et al. [11] found a CD rate of 45.5 % and excessive medical interventions during labor and vaginal delivery in low-risk women. Only 3.2 % of low-risk primiparous women had a natural vaginal childbirth and the public health services had the highest rate of use of good practices. The five good practices that were included in the composite adopted in this study—access to fluids or food, freedom to ambulate, use of non-pharmacological methods for pain relief, presence of a companionship, and use of partogram—are part of the recommendations of the Brazilian Ministry of Health [31] and WHO labor assistance guides [32].

Restriction of oral intake may be unpleasant for some women, and may adversely influence their experience of labor. In a systematic review of randomized controlled trials and quasi-randomized controlled trials, Singata et al. [33] concluded that the evidence shows no benefits or harm, and there is no justification for restriction of fluids and food in labor for women at low risk of complications.

Lavender et al. [34] in a systematic review of randomized and quasi-randomized controlled trials did not find any difference between use of a partogram and no partogram in the risk ratio of CD. However, when comparing the 3- and 4-h action line groups, the CD rate was lowest in the 4-h action line group. Additionally, when a partogram with a latent phase (composite) and one without (modified) were compared, the CD rate was lower in the partogram without a latent phase.

There is evidence that walking and upright positions in the first stage reduces the duration of labor, the risk of CD and the need for epidural. In a systematic review, Lawrence et al found that women who were upright during labor were also less likely to have CD (RR 0.71, 95 % CI 0.54 to 0.94) and less likely to have an epidural (RR 0.81, 95 % CI 0.66 to 0.99) [35]. Leal et al [11] found that in Brazil less than 50 % of low risk women who went into labor could ambulate. We found similar results in primiparous women.

Almost 10 % of primiparous women in our study had an epidural during labor and its use was associated with an increase in the rate of CD. The effect of epidural analgesia on the CD rate in the literature is controversial. Eriksen et al. [36] found that the use of epidural analgesia for labor pain was associated with higher risks of emergency CD and vacuum extraction. Other studies did not find any difference in the rate of CD with the use of epidural analgesia, but demonstrated that it is associated with a longer second-stage labor [37] and increased risk for an instrumental delivery [38].

The type of professional who provided care during labor was not associated with intrapartum CD in the adjusted analysis. This result is contrary to those observed in studies conducted in different settings [39, 40], including Brazil [41], and to the results of a systematic review that demonstrated that women who had midwife-led continuity models of care were more likely to experience spontaneous vaginal birth (average RR 1.05, 95 % CI 1.03 to 1.07) [42].

In our study, the presence of a nurse midwife was associated with increased use of good practices and less use of epidural analgesia, both conditions associated with a lower risk for CD. At a national level, only 18.7 % of primiparous women had access to a nurse midwife whilst being cared for labor. The highest rate was observed in the Southeast, the region that also presented the lowest rate of CD in the country. While future studies are necessary to confirm this hypothesis, it is possible that one explanation for the low rate of CD in the Southeast region is the significantly greater presence of a nurse midwife during labor care in SUS services located in this region. One study conducted in a health service located in this region [41] demonstrated lower rates of intrapartum CD in labor care provided by a team composed of nurse-midwife and obstetrician working collaboratively when compared to care provided by an obstetrician alone.

Social and demographic factors were not associated with intrapartum CD. Although we didn’t identify age as a risk factor for intrapartum CD, it is noteworthy that almost half of the primiparous women in our study were younger than 20 years. In the USA, in 2012, only 2.5 % of primiparous women were adolescent [43]. In a context of high proportions of young primiparous women, reducing the CD rate is even more important because a uterine scar will have repercussions on the reproductive lives of these young women. Delbaere et al [44] considered that if physicians want to stop the rising CD rate, they must concentrate on low-risk primiparous women. Reducing primary CD will also have an effect on repeat sections in the future.

This is the first national Brazilian study that has assessed labor and birth care with a sampling process that allows estimates for all macro-regions, location of service (capital and non-capital) and type of service (public, mixed and private). However, this study has some limitations. First, we conducted the study in institutions with more than 500 deliveries each year, which are responsible for almost 80 % of all deliveries [14]. The results presented here are not applicable to smaller hospitals that provide care to less than 500 births/year. Another limitation is the large amount of missing information (48 %) in the variable related to the linkage of the women to the maternity service. However, this occurred because only women who had received guidance provided by ANC on maternity reference for childbirth care answered this question. All other data had at least 89.6 % of information completed. Finally, we had limited information about the use of good practices during labor, as no information was available concerning the duration and time of use of each of the practices assessed. The same limitations apply to the presence of a nurse midwife during labor and the context of her assistance.

## Conclusion

The rate of CD in primiparous women in the public health services in Brazil is extremely high. Strategies for reducing the rate of CD and interventions in childbirth should focus on primiparous women as a priority. Reducing and adequately managing clinical and obstetric complications, counseling primiparous women on the advantages of a vaginal birth and supporting their decision for a vaginal birth can help to reduce this rate. Avoiding early admission, promoting the use of best practices during assistance with labor, inducing labor when indicated, and the judicious use of epidural analgesia when indicated can also reduce unnecessary intrapartum CD. Further research is necessary to determine the effects of labor care provided by a nurse midwife on the rates of intrapartum CD.

## Additional files

Additional file 1: Uma versão em português deste artigo está disponível no arquivo anexo 1. (DOCX 485 kb)
Additional file 2: A revisão dos colaboradores para este artigo estão disponíveis no arquivo anexo 2. (PDF 245 kb)

## Abbreviations

ANC: Antenatal care
CD: Cesarean delivery
SUS: Brazilian Public Health System
IOL: Induction of labor
WHO: World Health Organization

## References

[1] Betrán AP, Merialdi M, Lauer JA, Bing-Shun W, Thomas J, Van Look P (2007). Rates of caesarean section: analysis of global, regional and national estimates. Paediatr Perinat Epidemiol.

[2] Ye J, Betrán AP, Guerrero Vela M, Souza JP, Zhang J (2014). Searching for the optimal rate of medically necessary cesarean delivery. Birth.

[3] Villar J, Valladares E, Wojdyla D, Zavaleta N, Carroli G, Velazco A, WHO 2005 global survey on maternal and perinatal health research group (2006). Caesarean delivery rates and pregnancy outcomes: the 2005 WHO global survey on maternal and perinatal health in Latin America. Lancet.

[4] Timor-Tritsch IE, Monteagudo A (2012). Unforeseen consequences of the increasing rate of cesarean deliveries: early placenta accreta and cesarean scar pregnancy: a review. Am J Obstet Gynecol.

[5] Brasil. Ministério da Saúde. Secretaria de Vigilância em Saúde. Departamento de Análise de Situação de Saúde (2012). Saúde Brasil 2011: uma análise da situação de saúde e a vigilância da saúde da mulher/Ministério da Saúde, Secretaria de Vigilância em Saúde, Departamento de Análise de Situação de Saúde.

[6] SINASC - http://www2.datasus.gov.br/DATASUS/index.php?area=0205&VObj=http://tabnet.datasus.gov.br/cgi/deftohtm.exe?sinasc/cnv/nv. Accessed 10 Aug 2015.

[7] Gibbons L, Belizán JM, Lauer JA, Betrán AP, Merialdi M, Althabe F. The global numbers and costs of additionally needed and unnecessary caesarean sections performed per year: overuse as a barrier to universal coverage. World Health Report (2010) Background Paper, No 30.

[8] Dahlen HG, Tracy S, Tracy M, Bisits A, Brown C, Thornton C (2012). Rates of obstetric intervention among low-risk women giving birth in private and public hospitals in NSW: a population-based descriptive study. BMJ Open.

[9] Tracy SK, Welsh A, Hall B, Hartz D, Lainchbury A, Bisits A (2014). Caseload midwifery compared to standard or private obstetric care for first time mothers in a public teaching hospital in Australia: a cross sectional study of cost and birth outcomes. BMC Pregnancy Childbirth.

[10] Nippita TA, Lee YY, Patterson JA, Ford JB, Morris JM, Nicholl MC (2015). Variation in hospital caesarean section rates and obstetric outcomes among nulliparae at term: a population-based cohort study. BJOG.

[11] Carmo Leal MD, Pereira AP, Domingues RM, Theme Filha MM, Dias MA, Nakamura-Pereira M (2014). Obstetric interventions during labor and childbirth in Brazilian low-risk women. Cad Saude Publica.

[12] Zhang J, Troendle J, Reddy UM, Laughon SK, Branch DW, Burkman R, Consortium on Safe Labor (2010). Contemporary cesarean delivery practice in the United States. Am J Obstet Gynecol.

[13] Vasconcellos MTL, Silva PLN, Pereira APE, Schilithz AOC, Souza Junior PRB, Szwarcwald CL (2014). Desenho da amostra *Nascer no Brasil*: Pesquisa Nacional sobre Parto e Nascimento. Cad Saude Publica.

[14] do Carmo Leal M, da Silva AA, Dias MA, da Gama SG, Rattner D, Moreira ME, Filha MM, Domingues RM, Pereira AP, Torres JA, Bittencourt SD, D’orsi E, Cunha AJ, Leite AJ, Cavalcante RS, Lansky S, Diniz CS, Szwarcwald CL. Birth in Brazil: national survey into labour and birth. Reprod Health. 2012;9:15. doi:10.1186/1742-4755-9-15 10.1186/1742-4755-9-15 PMC3500713 22913663

[15] Victora CG, Huttly SR, Fuchs SC, Olinto MT (1997). The role of conceptual frameworks in epidemiological analysis: a hierarchical approach. Int J Epidemiol.

[16] Ministério da Saúde (2006). Pré-Natal e Puerpério: atenção qualificada e humanizada.

[17] American College of Obstetricians and Gynecologists (2014). Safe prevention of the primary cesarean delivery. Obstetric Care Consensus No. 1. Obstet Gynecol.

[18] Bryant AS, Washington S, Kuppermann M, Cheng YW, Caughey AB (2009). Quality and equality in obstetric care: racial and ethnic differences in caesarean section delivery rates. Paediatr Perinat Epidemiol.

[19] O’Neill SM, Agerbo E, Kenny LC, Henriksen TB, Kearney PM, Greene RA (2014). Cesarean section and rate of subsequent stillbirth, miscarriage, and ectopic pregnancy: a Danish register-based cohort study. PLoS Med.

[20] Domingues RMSM, Hartz ZMA, Dias MAB, Leal MC (2012). Avaliação da adequação da assistência pré-natal na rede SUS do Município do Rio de Janeiro, Brasil. Cad Saude Publica.

[21] Coutinho T, Monteiro MFG, Sayd JD, Teixeira MTB, Coutinho CM, Coutinho LM (2010). Monitoring the prenatal care process among users of the Unified Health Care System in a city of the Brazilian Southeast. Rev Bras Ginecol Obstet.

[22] Carroli G, Rooney C, Villar J (2001). How effective is an- tenatal care in preventing maternal mortality and serious morbidity? An overview of the evidence. Paediatr Perinat Epidemiol.

[23] Domingues RM, Dias MA, Nakamura-Pereira M, Torres JA, d’Orsi E, Pereira AP (2014). Process of decision-making regarding the mode of birth in Brazil: from the initial preference of women to the final mode of birth. Cad Saude Publica.

[24] Mealing NM, Roberts CL, Ford JB, Simpson JM, Morris JM (2009). Trends in induction of labour, 1998-2007: a population-based study. Aust N Z J Obstet Gynaecol.

[25] Recent declines in induction of labor by gestational age. NCHS Data Brief. No. 155. June 2014. http://www.cdc.gov/nchs/data/databriefs/db155.pdf. Accessed 10 Aug 2015. 24941926

[26] Wood S, Cooper S, Ross S (2014). Does induction of labour increase the risk of caesarean section? A systematic review and meta-analysis of trials in women with intact membranes. BJOG.

[27] Mishanina E, Rogozinska E, Thatthi T, Uddin-Khan R, Khan KS, Meads C (2014). Use of labour induction and risk of cesarean delivery: a systematic review and meta-analysis. CMAJ.

[28] Holmes P, Oppenheimer L, Wen S (2001). The relationship between cervical dilatation at initial presentation in labour and subsequent intervention. Br J Obstet Gynaecol.

[29] Janssen PA, Weissinger S (2014). Women’s perception of pre-hospital labour duration and obstetrical outcomes; a prospective cohort study. BMC Pregnancy Childbirth.

[30] Neal JL, Lamp JM, Buck JS, Lowe NK, Gillespie SL, Ryan SL (2014). Outcomes of nulliparous women with spontaneous labor onset admitted to hospitals in preactive versus active labor. J Midwifery Womens Health.

[31] Secretaria de Políticas de Saúde, Ministério da Saúde (2001). Parto, aborto e puerpério: assistência humanizada à mulher.

[32] World Health Organization, Maternal and Newborn Health/Safe Motherhood Unit (1996). Care in normal birth: a practical guide.

[33] Singata M, Tranmer J, Gyte GM (2010). Restricting oral fluid and food intake during labour. Cochrane Database Syst Rev.

[34] Lavender T, Hart A, Smyth RM (2012). Effect of partogram use on outcomes for women in spontaneous labour at term. Cochrane Database Syst Rev.

[35] Lawrence A, Lewis L, Hofmeyr GJ, Styles C. Maternal positions and mobilityduring first stage labour. Cochrane Database Syst Rev. 2013;(10):CD003934. doi:10.1002/14651858.CD003934. 10.1002/14651858.CD003934.pub4 PMC11664456 24105444

[36] Eriksen LM, Nohr EA, Kjaergaard H (2011). Mode of delivery after epidural analgesia in a cohort of low-risk nulliparas. Birth.

[37] Leighton BL, Halpern SH (2002). The effects of epidural analgesia on labor, maternal, and neonatal outcomes: a systematic review. Am J Obstet Gynecol.

[38] Anim-Somuah M, Smyth RM, Jones L (2011). Epidural versus non-epidural or no analgesia in labour. Cochrane Database Syst Rev.

[39] McLachlan HL, Forster DA, Davey MA, Farrell T, Gold L, Biro MA (2012). Effects of continuity of care by a primary midwife (caseload midwifery) on caesarean section rates in women of low obstetric risk: the COSMOS randomised controlled trial. BJOG.

[40] Wong N, Browne J, Ferguson S, Taylor J, Davis D (2015). Getting the first birth right: A retrospective study of outcomes for low-risk primiparous women receiving standard care versus midwifery model of care in the same tertiary hospital. Women Birth.

[41] Vogt SE, da Silva KS, Dias MAB (2014). Comparison of childbirth care models in public hospitals, Brazil. Revista de Saude Publica.

[42] Sandall J, Soltani H, Gates S, Shennan A, Devane D (2016). Midwife-led continuity models versus other models of care for childbearing women. Cochrane Database Syst Rev.

[43] National vital statistics report births: final data for 2013. http://www.cdc.gov/nchs/data/nvsr/nvsr64/nvsr64_01.pdf. Accessed 10 Aug 2015. 25603115

[44] Delbaere I, Cammu H, Martens E, Tency I, Martens G, Temmerman M. Limiting the caesarean section rate in low risk pregnancies is key to lowering the trend of increased abdominal deliveries: an observational study. BMC Pregnancy Childbirth. 2012;12:3. 10.1186/1471-2393-12-3 PMC3267690 22230339

