# Supplementary material for: Factors associated with cesarean delivery during labor in primiparous women assisted in the Brazilian Public Health System: data from a National Survey
Source: Reprod Health. 2016 Oct 17;13(Suppl 3):114. doi: 10.1186/s12978-016-0231-z (PMC5073796; doi:10.1186/s12978-016-0231-z)
Supplement: Additional file 1: — Uma versão em português deste artigo está disponível no arquivo anexo 1. (DOCX 485 kb) [file 12978_2016_231_MOESM1_ESM.docx]

Fatores associados à realização de cesariana intraparto em primíparas assistidas no Sistema Único de Saúde: Dados de um estudo nacional.

Marcos Augusto Bastos Dias^1^*, Rosa Maria Soares Madeira Domingues^2^, Arthur Orlando Corrêa Schilithz^3^, Marcos Nakamura-Pereira^1^ e Maria do Carmo Leal^3^.

1. Instituto Fernandes Figueira/FIOCRUZ

Av. Rui Barbosa 716, Rio de Janeiro, Brasil. CEP: 22250-020

2. Instituto Nacional de Infectologia Evandro Chagas/FIOCRUZ

Av. Brasil, 4365 - Manguinhos, Rio de Janeiro, Brasil. CEP: 21040-360

3. Escola Nacional Saúde Publica Sérgio Arouca/FIOCRUZ

Av. Brasil, 4365 - Manguinhos, Rio de Janeiro, Brasil. CEP: 21040-360

*Autor para correspondência: Marcos Augusto Bastos Dias

E-mail: [marcosad@iff.fiocruz.br](mailto:marcosad@iff.fiocruz.br)

Rosa Domingues [rosamsmd@gmail.com](mailto:rosamsmd@gmail.com)

Arthur Schilithz [artmestre@gmail.com](mailto:artmestre@gmail.com)

Marcos Nakamura-Pereira [marcosnakamura@globo.com](mailto:marcosnakamura@globo.com)

Maria do Carmo Leal duca@ensp.fiocruz.br

Resumo

Introdução

A taxa de cesariana no Brasil tem aumentado nos últimos 40 anos. Essa taxa no serviço público é três vezes maior do que a recomendada pela Organização Mundial da Saúde (OMS). Entre as estratégias para reduzir a taxa de cesarianas, a mais importante é a redução da taxa de cesariana primária. Este estudo tem como objetivo descrever os fatores associados à realização da cesariana durante o trabalho de parto em primíparas com gestação única em apresentação cefálica atendidas no Sistema Único de Saúde (SUS).

Métodos

Este estudo é parte do inquérito Nascer no Brasil, um estudo nacional de base hospitalar com 23,894 puérperas e seus recém-nascidos. A taxa de cesariana em primíparas foi estimada. Analises univariadas e multivariadas com regressão logística foram realizadas para analisar os fatores associados à realização de cesariana durante o trabalho de parto em primíparas com gestação única em apresentação cefálica, incluindo as estimativas brutas e ajustadas das razões de risco e seus intervalos de confiança de 95%.

Resultados

Os dados analisados são relativos às 2814 primíparas elegíveis que tiveram um parto vaginal ou uma cesariana durante o trabalho de parto em hospitais do SUS. Na análise ajustada residir na região sudeste esteve associada com menor taxa de cesariana durante o trabalho de parto. A ocorrência de condições clinicas ou obstétricas potencialmente relacionadas às emergências obstétricas antes do nascimento, a admissão precoce com < de 4 cm de dilatação, a decisão no final da gestação por uma cesariana e o uso de analgesia estiveram associados com maior risco de cesariana. Aconselhamento favorável ao parto vaginal durante o pré-natal, indução do trabalho de parto e o uso de alguma das boas práticas durante o trabalho de parto foram fatores protetores para a cesariana. O tipo de profissional que assistiu ao parto não teve significância na análise final, mas a análise bivariada mostrou uma maior proporção do uso das boas práticas e uma menor proporção de analgesia epidural em mulheres que receberam cuidado de pelo menos uma enfermeira obstétrica.

Conclusões

A taxa de cesariana em primíparas no SUS no Brasil é extremamente alta e pode comprometer a saúde destas mulheres e de seus bebês. Informação e suporte para o parto normal durante o pré-natal, evitar a admissão precoce e promover o uso das boas práticas durante a assistência ao trabalho de parto podem reduzir as cesarianas desnecessárias.

Palavras chave: cesariana, primíparas, assistência ao trabalho de parto, enfermeira obstétrica, boas práticas.

Introdução

Na obstetrícia moderna a cesariana como uma forma segura de nascimento é responsável pela redução da mortalidade materna e neonatal [1]. Entretanto, Ye et al. [2] demostraram que um aumento na taxa de cesariana acima de 10% não apresenta benefícios para a mulher ou para o recém-nascido e pode estar associada com complicações maternas e neonatais e aumentar os custos do sistema do saúde [3]. Cesarianas desnecessárias podem comprometer o futuro reprodutivo das mulheres [4].

A taxa de cesariana tem aumentado de maneira constante no Brasil nos últimos 40 anos e subiu de 38% em 2000 para 50.6% em 2009 quando esta cirurgia se tornou a principal forma de nascimento no país [5]. Em 2013, 56.6% dos nascimentos foram por cesarianas [6]e um estudo realizado pela Organização Mundial de Saúde (OMS) estimou que no Brasil em 2008 ocorreram aproximadamente 1.000.000 de cesarianas desnecessárias [7].

No Brasil, embora a taxa de cesariana nos hospitais privados seja a mais elevada, nos serviços públicos ela é aproximadamente três vezes maior do que as taxas recomendadas [4]. O Sistema Único de Saúde (SUS) é financiado pelo governo, livre de quaisquer custos e teve uma taxa de cesariana de 37,1% em 2010. O sistema privado participa de forma complementar, é responsável por quase 20 % de todos os nascimentos e teve uma taxa de cesariana de 63,6% no mesmo ano [5].

O modelo obstétrico de atenção ao parto tem sido identificado como uma das causas do aumento das taxas de cesariana [8-10]. Nos hospitais brasileiros, o uso das boas práticas na atenção ao parto é ainda muito baixo, apesar das evidências de que essas práticas estão associadas à menores taxas de intervenção, maior satisfação materna e bons resultados perinatais [11].

Em primíparas, cujos trabalhos de parto e parto tendem a ser mais longos, a realização de cesariana e outros resultados maternos e perinatais estão diretamente relacionados à qualidade de cuidado. Estudo de Leal et al. [11] mostrou que, no Brasil primíparas são admitidas precocemente e estão mais expostas às intervenções hospitalares durante o trabalho de parto e o parto quando comparadas a multíparas. Diferentes autores têm enfatizado o papel central da redução das cesarianas primárias para a redução das taxas de cesarianas [12].

O objetivo deste estudo foi estimar a taxa de cesariana eletiva e intraparto em mulheres primíparas com uma gestação única com feto em apresentação cefálica assistidas nos serviços do sistema único de saúde (SUS) e descrever os fatores associados à cesariana durante a assistência ao trabalho de parto e parto.

Métodos

Este estudo é parte do Inquérito Nascer no Brasil, um estudo nacional de base hospitalar que avaliou uma amostra de mulheres puérperas e seus recém-nascidos realizada entre fevereiro de 2011 e Outubro de 2012. Hospitais com 500 ou mais partos anuais foram selecionados e estratificados de acordo com a região, localização (capital do estado ou interior) e tipo de serviço de saúde (público, privado ou misto). Um total de 23.894 puérperas foram entrevistadas em 266 hospitais distribuídos em todos os estados brasileiros. Maiores detalhes sobre o desenho da amostra foram publicados por Vasconcellos et al. [13]. Na primeira etapa do estudo entrevistas face a face foram realizadas com puérperas durante sua hospitalização e dados foram coletados dos prontuários maternos e neonatais. Cartões pré-natal forma fotografados. Mais detalhes sobre a coleta de dados foram publicados por do Carmo Leal et al. [14].

Foram analisados dados sobre primíparas com gestação única e feto em apresentação cefálica nascidos vivos ou natimortos com mais de 22 semanas de gestação, assistidos no SUS. Mulheres que se auto declaravam como amarelas ou indígenas foram excluídas desta análise pela pequena proporção destas participantes na amostra (1,1% e 0,4% da amostra total respectivamente). Análise univariada e regressão logística não condicional dos fatores associados à cesariana no trabalho de parto em primíparas assistidas no SUS foram feitas utilizando um modelo teórico com três níveis hierárquicos [15] (Figura 1). No nível distal foram incluídas variáveis sociodemográficas maternas: “idade materna” (12-19 anos, 20-34 anos, ou ≥ 35 anos); “escolaridade” (até 7 anos, 8-10 anos, 11-14 anos e ≥ 15 anos de escolaridade); “cor da pele auto-referida” (branca, preta ou parda); “trabalho remunerado” (sim ou não); “situação conjugal” (vive com ou sem parceiro) e macro região do país (Norte, Sul, Nordeste, Sudeste e Centro-Oeste). No nível intermediário, foram incluídas variáveis relacionadas às características da gestação, realização de pré-natal (PN) e os serviços de atenção ao parto: “adequação do pré-natal” (considerando um mínimo de sete consultas e rotina de exames para uma gestação a termo, ajustado para a idade gestacional ao nascimento de acordo com o protocolo do Ministério da Saúde [16]); “complicações clínicas ou obstétricas potencialmente associadas a emergências obstétricas antes do nascimento” (placenta prévia, descolamento placentário, síndromes hipertensivas, diabetes, infecção materna e infecção pelo HIV); “mulheres atendidas na maternidade de referência” (se a mulher foi atendida no momento do parto na maternidade de referência segundo orientação pré-natal, sim ou não); “procura por vaga na maternidade antes da admissão hospitalar” (procura por mais de uma maternidade durante o trabalho de parto, sim ou não); “aconselhamento pré-natal favorável ao parto vaginal” (sim ou não); e “decisão pelo tipo de parto no final da gestação” (se ao final da gestação, durante o pré-natal, a mulher já havia decido pelo tipo de parto; não, sim parto vaginal ou sim cesariana). No nível proximal foram incluídas as variáveis associadas ao trabalho de parto: “trabalho de parto espontâneo ou induzido”; “o mesmo profissional prestou assistência pré-natal e ao trabalho de parto e parto” (sim ou não); “dilatação cervical na admissão (< 4 cm – admissão precoce ou ≥ 4 cm); “uso de alguma boa prática durante o trabalho de parto” (dieta, movimentação, uso de métodos não farmacológicos para alívio da dor, presença de acompanhante e monitoramento da evolução do trabalho de parto com o uso de partograma); “presença de pelo menos uma enfermeira obstétrica durante a assistência ao trabalho de parto” (sim ou não); “uso de cateter venoso”(sim ou não); e “uso de analgesia raquidiana ou epidural” (sim ou não).

O resultado principal foi o tipo de parto (parto vaginal ou cesariana durante o trabalho de parto). As cesarianas foram classificadas segundo informação de prontuários hospitalares sendo consideradas como cesarianas “ durante o trabalho de parto” se a mulher teve um trabalho de parto espontâneo ou induzido e foi submetida a uma cesariana com dilatação cervical de pelo menos 4 cm [17]. Todas as outras cesarianas foram classificadas como eletivas independentemente da duração do trabalho de parto ou a indicação da cesariana. As variáveis “complicações clínicas ou obstétricas potencialmente associadas a emergências obstétricas antes do nascimento”, “trabalho de parto espontâneo ou induzido”, “dilatação cervical na admissão”, “monitoramento da evolução do trabalho de parto com o partograma” , “presença de pelo menos uma enfermeira obstétrica durante a assistência ao trabalho de parto”, “uso de cateter venoso”, “uso de analgesia raquidiana ou peridural” e “tipo de parto” foram baseadas nas informações de prontuários hospitalares. Todas as outras variáveis foram informadas pelas mulheres durante a entrevista. Partos à fórceps foram analisados juntos com outros nascimentos vaginais por seu pequeno número (1%). Não foram observados partos com o uso de vácuo-extrator.

Análise univariada foi utilizada para estimar as razões de chance não ajustadas (RC) e intervalo de confiança de 95% (IC 95%). No primeiro modelo multivariado todas as variáveis distais foram incluídas. Variáveis do nível distal com um nível de significância estimado menor que 0,20 foram incluídas no segundo modelo, que também incluiu todas as variáveis do nível intermediário. No terceiro modelo, variáveis distais e intermediárias com um nível de significância estimado menor que 0,20 e as variáveis proximais foram incluídas. Todas as variáveis com um nível de significância <0.05 e algumas variáveis de interesse de acordo com o modelo teórico foram mantidas no modelo multivariado final. Os resultados do modelo multivariado final estão expressos como razões de chance ajustadas com seus respectivos IC de 95%.

Para analisar a associação do uso de boas práticas durante o trabalho de parto e o uso de analgesia raquidiana ou peridural ao profissional que prestou assistência ao trabalho de parto foi utilizado o teste de qui-quadrado para verificar as diferenças entre proporções com um nível de significância de <0.05.

Em todas as analises estatísticas levou-se em consideração o processo de amostragem complexa utilizado. A ponderação dos dados foi calculada de acordo com o inverso da probabilidade da inclusão de cada puérpera na amostra. Para garantir que a distribuição das puérperas que foram entrevistadas fosse similar àquela observada entre os nascimentos na população da amostra em 2011, procedimentos de calibração foram utilizados na seleção de cada estrato [13]. Os procedimentos de ponderação e calibração foram utilizados para todos os dados (números e proporções) apresentados neste estudo.

As análises foram realizadas utilizando-se o software IBM SPSS versão 20.0 (IBM Corp., Armonk, USA).

Este estudo foi aprovado pelo Comitê de Ética em Pesquisa da ENSP/FIOCRUZ com o número 92/2010. Foram tomadas medidas para garantir a privacidade e a confidencialidade dos dados coletados com as mulheres. Consentimento informado foi obtido antes das entrevistas com o uso de um formulário para consentimento informado.

Resultados

Das 23.894 mulheres entrevistadas no inquérito Nascer no Brasil, 9.838 (41,2%) foram atendidas em hospitais do SUS. Do total de 4.298 primíparas que tiveram parto em hospitais do SUS, 4.079 (96,1%) tinham uma gestação única com feto em apresentação cefálica. Destas, 1.592 (39%) foram submetidas à cesariana, a maioria (1134, 27,8%) antes do início do trabalho de parto e 131 (3,2%) tiveram uma indicação para cesariana no momento da admissão hospitalar. O número de primíparas com uma gestação única em apresentação cefálica que tiveram uma indicação para cesariana durante o trabalho de parto foi 327 (11,6% das mulheres elegíveis). Os dados analisados são relativos às 2.814 primíparas com gestação única em apresentação cefálica que tiveram um parto vaginal ou uma cesariana durante o trabalho de parto em hospitais do SUS. A figura 2 sintetiza a distribuição das gestantes que foram atendidas em hospitais do SUS.

Quase metade (47,7%)das entrevistadas tinha até 19 anos de idade, 51% tinham entre 20 e 34 anos e apenas 1% tinham 35 anos ou mais. A maioria (65,2%) das mulheres se auto-declarou parda, tinha até 8 anos de estudo (58,1%), vivia com o parceiro (71,9%) e não tinha trabalho remunerado (73,7%). A maioria das primíparas vivia na região Nordeste (37,7%), seguida pela região Sudeste (36,6%), Norte (12,7%), Sul (6,7%) e Centro-Oeste (6,3%).

Quase todas (99%) as primíparas tiveram acompanhamento pré-natal, enquanto somente 7,2% tiveram acompanhamento pré-natal considerado adequado, quando avaliado segundo os critérios propostos no protocolo nacional [15]. A maioria (77%) das primíparas informou que foi assistida na maternidade de referência e 19.4% teve que procurar por um serviço de atenção ao parto antes da admissão hospitalar. Condições clínicas e obstétricas potencialmente relacionadas a emergências obstétricas antes do parto estiveram presentes em 13,2% das primíparas e 4,5% delas já havia optado por uma cesariana no final da gestação. Aconselhamento favorável ao parto vaginal durante a assistência pré-natal foi observado em 72,1 % das primíparas e 61,2% tinha optado por um parto vaginal no final da gravidez. Aproximadamente 17% das primíparas teve seu trabalho de parto induzido, 1,9% foi assistida pelo mesmo profissional durante a gestação e o parto e 37,6% teve admissão precoce na maternidade com < de 4cm de dilatação. Durante a assistência ao trabalho de parto, 85% das primíparas teve acesso à pelo menos uma das boas práticas (30,4% recebeu líquidos ou alimentos, 48% pode deambular, 36,4% usou algum método não farmacológico para alívio da dor, em 52,6% um partograma foi utilizado para monitorar o trabalho de parto e 56,4% teve um acompanhante de sua escolha). Um cateter venoso foi colocado em 67,9% das primíparas, 9,3% fez uso de analgesia e 18,7% foi assistida por pelo menos uma enfermeira obstétrica durante o trabalho de parto.

Dentre todos os fatores distais analisados, somente a região de residência da mulher mostrou uma diferença significativa para o desfecho principal “cesariana durante o trabalho de parto”. Primíparas vivendo na região Sudeste tiveram uma taxa de cesariana de 6,7%, seguidas pelas residentes na região Nordeste com 13,9%, 14% na região Norte, 15,3% na região Sul e a maior taxa de 18,6% na região Centro-Oeste (Tabela 1).

No nível intermediário, dentre as características relacionadas à gestação atual, as condições clínicas e obstétricas relacionadas a emergências obstétricas e a decisão por uma cesariana no final da gestação mostraram associação com maior ocorrência de cesariana durante o trabalho de parto. Aconselhamento favorável ao parto vaginal foi protetora para esta ocorrência. Adequação do pré-natal, ter sido assistida na maternidade de referência, e a procura por mais de um serviço para assistência ao trabalho de parto e parto não mostraram associação significativa com cesariana durante o trabalho de parto (Tabela 1).

Entre os fatores relacionados com a assistência no trabalho de parto (Tabela 1), ter trabalho de parto induzido, usar pelo menos uma boa prática durante o trabalho de parto, e a presença de pelo menos uma enfermeira obstétrica durante o trabalho de parto foram fatores protetores para a ocorrência de cesariana. Admissão hospitalar precoce com dilatação cervical < 4 cm, e o uso de analgesia durante o trabalho de parto estiveram associados com maior ocorrência de cesariana durante o trabalho de parto. Ter tido o mesmo profissional para a assistência pré-natal e para o parto e o uso de cateter venoso não mostraram associação significativa com o desfecho principal.

Os resultados da regressão logística multivariada (Tabela 2) mostraram que, entre as variáveis distais, somente a região de residência manteve significância estatística, com um risco aumentado de cesariana durante o trabalho de parto para as regiões Nordeste (RC 2,3; IC 95% 1,1–4,7), Norte (RC 2,5; IC 95% 1,3–4,6), Sul (RC 2,8; IC 95% 1,4–5,5), e Centro-Oeste (RC 3,4; IC 95% 1,6–7,1) quando comparadas à região Sudeste. A ocorrência de condições clínicas e obstétricas potencialmente relacionadas a emergências obstétricas antes do nascimento (RC 2,7; IC 95% 1,7–4,4), admissão precoce com < 4cm de dilatação (RC 2,1; IC 95% 1,4 – 3,3), a decisão no final da gestação por uma cesariana (RC 12,1; IC 95% 7,1-20,7) e o uso de analgesia (RC 3,7; IC 95% 1,8-7,6 ) estiveram associados a maior risco de cesariana durante o trabalho de parto. Aconselhamento favorável ao parto vaginal durante o pré-natal (RC 0,5; IC 95% 0,3-0,6), indução do trabalho de parto (RC 0,2; IC 95% 0,1-0,4), e o uso de pelo menos uma boa prática durante o trabalho de parto (RC 0,3; IC 95% 0,2-0,5) foram fatores protetores para a realização de cesariana.

O tipo de profissional que assistiu o nascimento não foi significativo na análise final (RC 1,0; IC 95% 0,6-1,7), mas a variável foi mantida no modelo final por sua relevância. Na análise bivariada, mulheres que tiveram a presença de pelo menos uma enfermeira obstétrica durante o trabalho de parto tiveram maior utilização de boas práticas e uma menor proporção do uso de analgesia epidural (Tabela 3). Uma maior proporção de enfermeiras obstétricas atuando na assistência ao trabalho de parto também foi observada na região Sudeste (36,9%, dado não mostrado em tabela), região do país com menor taxa de cesariana.

Discussão

Este estudo estimou a taxa de cesariana eletiva (27,8%) e durante o trabalho de parto (8%) em primíparas com uma gestação única em apresentação cefálica assistidas nos serviços públicos no Brasil. A taxa total de cesariana de 35,8% é alta quando comparada a estudos com populações semelhantes. Bryant et al. [18] encontraram uma taxa de cesariana de 17,8% em primíparas de baixo risco numa coorte retrospectiva de mulheres que tiveram seus partos na Universidade da Califórnia, São Francisco, entre 1980 e 2001, e O`Neill et al [19] identificaram uma taxa de 12,5% de cesariana de emergência e 4,68% de cesariana eletiva, entre 1982 e 2010, numa coorte de nascidos vivos em primíparas na Dinamarca.

A maioria das cesarianas em nosso estudo foram eletivas, mas nesta análise foram identificados os fatores associados à cesariana durante o trabalho de parto. A ocorrência de condições clinicas e obstétricas potencialmente relacionadas a emergências obstétricas antes do nascimento, a decisão no final da gestação por uma cesariana, admissão precoce com < 4 cm e o uso de analgesia estiveram associados à uma maior proporção de cesarianas. Residir na região Sudeste, aconselhamento favorável ao parto normal durante o pré-natal, indução do trabalho de parto e o uso de qualquer boa prática durante o trabalho de parto foram fatores protetores para a realização de cesariana.

Condições clínicas e obstétricas são fatores de risco conhecidos para a cesariana, seja eletiva ou durante o trabalho de parto [18,19]. As indicações de cesariana e o manejo das condições clinicas e obstétricas durante o cuidado pré-natal e no trabalho de parto não foram analisadas neste estudo. O Brasil tem uma alta cobertura de assistência pré-natal e em nosso estudo quase todas as primíparas tiveram pelo menos uma consulta pré-natal. Entretanto, menos de 10% teve um cuidado pré-natal adequado ou mais do que adequado quando avaliado segundo protocolos nacionais. Outros autores [20,21] também relataram elevada inadequação do cuidado pré-natal no Brasil. Esta inadequação pode resultar em resultados adversos já que muitas práticas utilizadas na rotina do cuidado pré-natal estão associadas a menores taxas de mortalidade materna e perdas fetais [22]. Em nosso estudo, o cuidado pré-natal não esteve associado à realização de cesariana durante o trabalho de parto, o que pode ser decorrente de um confundimento residual, já que o escore de adequação utilizado não avaliou o manejo de condições específicas.

O cuidado pré-natal não é importante apenas para o cuidado adequado de complicações obstétricas e clínicas. Nossos resultados mostraram que o aconselhamento favorável ao parto vaginal e a decisão por um parto vaginal ao final da gestação estiveram associados a uma menor taxa de cesariana durante o trabalho de parto. Domingues et al. [23] encontraram que no setor publico no Brasil as mulheres não são apoiadas em sua escolha por um parto vaginal porque sua preferencia por este tipo de parto diminui ao longo da gestação. O medo da dor no trabalho de parto é a principal razão para as mulheres preferirem uma cesariana no Brasil e a falta de suporte durante a gestação pode desencorajar as mulheres à medida que o parto vai ficando mais próximo.

A indução do trabalho de parto esteve associada a uma menor taxa de cesariana durante o trabalho de parto. Este é um procedimento comum nos diferentes países e pode modificar os resultados maternos e perinatais [24]. A taxa de indução do trabalho de parto em nosso estudo foi de 17,4% e é menor do que a encontrada por outro autores em populações similares: maior do que 23% para todas as gestações de feto único nos EUA em 2010 [25]; 29% em mulheres com 32 ou mais semanas de gestação em 2007 na Austrália [24]. Uma possível explicação para esta menor taxa de indução é a elevada taxa de cesariana eletiva encontrada em nosso estudo. Em estudos com gestações de alto e baixo risco [26] ou gestações à termo e pós-termo [27], indução do trabalho de parto estave associada com menores taxas de cesariana do que a conduta expectante.

A admissão precoce com < 4cm de dilaação é um risco conhecido para a cesariana [28] e ocorreu em 37.6% das mulheres em nossa amostra. Mulheres com admissão hospitalar na fase latente (<3 cm de dilatação) apresentaram maior risco de intervenções obstétricas, incluindo monitoramento eletrônico fetal contínuo, analgesia epidural, ocitocina e cesariana, do que aquelas admitidas na fase ativa do trabalho de parto [29]. Neal et al. [30] sugeriu uma rotina baseada em evidencias para admissão em trabalho de parto visandoreduzir a admissão inadvertida de mulheres na fase latente: quando não se consegue diagnosticar o trabalho de parto ativo com relativa certeza, a observação antes da internação deve ser garantida.

O uso de qualquer boa prática durante o trabalho de parto esteve associado com menores taxas de cesariana. No estudo Nascer no Brasil, Leal et al. [11] encontraram uma taxa de 45,5% e uso excessivo de intervenções médicas durante o trabalho de parto e parto vaginal em mulheres de baixo risco. Somente 3,2% das primíparas de baixo risco tiveram um parto vaginal natural e os serviços públicos tiveram a maior taxa de utilização das boas práticas. As cinco boas práticas que foram incluídas neste estudo – acesso a líquidos e alimentos, liberdade de deambulação, uso de métodos não farmacológicos de alivio da dor, presença de um acompanhante e uso do partograma – são parte das recomendações do Ministério da Saúde do Brasil [31] e dos guias de assistência ao trabalho de parto da OMS [32].

A restrição da ingesta oral pode ser desagradável para algumas mulheres e pode influenciar de modo adverso sua experiência do trabalho de parto. Numa revisão sistemática de estudos randomizados e quase-randomizados, Singata et al. [33] concluíram que as evidências não mostravam benefícios ou danos e que não há justificativa para a restrição de fluidos ou alimentos no trabalho de parto em mulheres com baixo risco de complicações.

Lavander et al [34] numa revisão sistemática de estudos randomizados e quase-randomizados não encontrou diferenças associadas ao uso do partograma na taxa de cesariana. Entretanto, quando partogramas com linha de ação em 3h e 4h foram comparados, a taxa de cesariana foi menor naqueles com linha de ação de 4h. Além disso, menor taxa de cesariana foi observada em partograma modificado, sem fase latente, quando comparado ao partograma padrão com fase latente.

Há evidência de que deambular e adotar posições verticais no primeiro estágio reduzem a duração do trabalho de parto, o risco de cesariana e a necessidade de epidural. Numa revisão sistemática, Lawrence et al. [43] encontraram que mulheres que ficaram nas posições verticais tinham menos chance de ter uma cesariana (RC 0,71, IC 95% 0,54 a 0,94) e menor chance de ter uma epidural (RC 0,81, IC 95% 0,66 a 0,99).

Leal et al. [11] encontraram que no Brasil menos de 50% das mulheres de baixo risco que entraram em trabalho de parto puderam deambular. Resultados similares foram observados em primíparas neste estudo.

Quase 10% das primíparas em nosso estudo receberam analgesia epidural durante o trabalho de parto e seu uso esteve associado à maior taxa de cesariana. O efeito da analgesia epidural na taxa de cesariana é controverso. Eriksen et al. [35] encontraram associação entre o uso da analgesia epidural no trabalho de parto e maior risco de cesariana de emergência e de uso de vácuo-extrator. Outros estudos não encontraram diferença na taxa de cesariana associada ao uso de analgesia epidural, mas demonstraram sua associação com uma segunda fase do trabalho de parto mais longa [36] e um risco maior de parto instrumental [37].

Em nosso estudo, na análise ajustada, o tipo de profissional que prestou assistência ao trabalho de parto não esteve associado com cesariana no trabalho de parto. Este resultado vai de encontro àquele observado em estudos realizados em diferentes locais [38,39], incluindo o Brasil [40], e aos resultados de uma revisão sistemática que demonstrou que mulheres que foram assistidas em modelos de atenção com cuidado continuo por obstetrizes apresentaram maior chance de parto vaginal espontâneo (RC1,05, IC 95% 1,03 a 1,07) [41].

Em nosso estudo, a presença da enfermeira obstetra esteve associada à maior uso de boas práticas e menor uso de analgesia epidural, ambas condições associadas com um menor risco de cesariana. A nível nacional somente 18.7% das primíparas tiveram acesso a uma enfermeira obstétrica durante seu trabalho de parto. A maior proporção foi observada no Sudeste, a região que também apresentou a menor taxa de cesariana no país. Embora estudos futuros sejam necessários para confirmar esta hipótese, é possível que uma das explicações para a menor taxa de cesariana na região Sudeste seja a presença significativamente maior de enfermeiras obstétricas nos serviços localizados nesta região. Um estudo realizado em serviços de saúde localizados nesta região [40] demonstrou taxas mais baixas de cesariana durante o trabalho de parto quando a assistência era realizada por um time composto por enfermeiras obstetras e obstetras trabalhando de forma colaborativa quando comparado com a assistência realizada apenas por obstetras.

Fatores sociais e demográficos não estiveram associados com a cesariana durante o trabalho de parto. Embora não tenhamos identificado a idade como um fator de risco para a cesariana durante o trabalho de parto cabe ressaltar que quase metade das primíparas em nosso estudo tinham menos que 20 anos de idade. Nos EUA em 2012, somente 2,5% das primíparas eram adolescentes [42]. Em um contexto com alta proporção de primíparas jovens reduzir as taxas de cesariana é ainda mais importante porque uma cicatriz uterina terá repercussões na vida reprodutiva dessas jovens mulheres. Segundo Delbare et al. [43], se os médicos desejam interromper o aumento da taxa de cesariana eles devem se concentrar nas mulheres primíparas de baixo risco. Reduzir a taxa primaria de cesariana também terá um efeito nas cesarianas repetidas no futuro.

Este é o primeiro estudo brasileiro que avaliou a assistência ao trabalho de parto e parto com uma amostragem que permite estimativas para todas as macrorregiões, localização do serviço (capital ou interior) e tipo de serviço (público, privado ou misto). Entretanto, esse estudo tem algumas limitações. Foram incluídos apenas hospitais com mais de 500 partos por ano, que são responsáveis por quase 80% de todos os nascimentos [44]. Os resultados apresentados aqui não são aplicáveis aos hospitais que realizam menos de 500 partos por ano. Outra limitação é a grande proporção de casos sem informação para a variável relacionada à vinculação da mulher a uma maternidade de referência. Entretanto, isto aconteceu porque apenas as mulheres que receberam orientação no pré-natal sobre a maternidade de referência para o parto responderam esta questão. Todos os outros dados tiveram pelo menos 89,6% de completude. Finalmente a informação sobre o uso de boas práticas durante o trabalho de parto é limitada, uma vez que nenhuma informação sobre a duração e momento do uso das práticas foi avaliada. As mesmas limitações se aplicam a presença da enfermeira obstétrica durante a assistência ao trabalho de parto e o contexto de sua atuação.

Conclusão

A taxa de cesariana em primíparas nos serviços públicos de saúde no Brasil é extremamente elevada. Estratégias para reduzir a taxa de cesariana e o uso de intervenções no parto devem priorizar as primíparas. Reduzir e manejar de forma adequada complicações clinicas e obstétricas, aconselhar primíparas sobre as vantagens do parto vaginal e apoiar sua decisão pelo parto vaginal podem ajudar a reduzir esta taxa. Evitar a admissão precoce, promover o uso das boas praticas durante a assistência ao trabalho de parto, induzir o trabalho de parto quando indicado e o uso judicioso da analgesia epidural quando indicado também podem reduzir a cesariana desnecessária durante o trabalho de parto. Pesquisas futuras são necessárias para determinar os efeitos da assistência ao trabalho de parto prestada por enfermeiras obstétricas nas taxas de cesariana no trabalho de parto.

Lista de abreviaturas

SUS Sistema Único de Saúde

OMS Organização Mundial de Saúde

PN Assistência pré-natal

Conflito de interesses

Os autores declaram não ter quaisquer conflitos de interesses.

Contribuição dos autores

MABD e RMSMD fizeram contribuições substanciais à concepção e desenho do estudo, coleta de dados e redigiram o manuscrito. MABD, RMSMD, MNP e AOCS fizeram a analise dos dados e revisaram criticamente os manuscritos. MCL fez contribuições substanciais ao desenho do estudo e produção dos dados. Todos os autores leram e aprovaram o manuscrito final.

Agradecimentos

Este trabalho teve o financiamento do Conselho Nacional de Desenvolvimento Cientifico e Tecnológico – CNPq, da Escola Nacional de saúde Publica, Fundação Oswaldo Cruz (Projeto INOVA) e Fundação de Amparo à Pesquisa do Estado do Rio de Janeiro (FAPERJ).

Referencias

1 - Betrán AP, Merialdi M, Lauer JA, Bing-Shun W, Thomas J, Van Look P, et al. Rates of caesarean section: analysis of global, regional and national estimates. Paediatr Perinat Epidemiol. 2007;21:98-113.

2 - Ye J, Betrán AP, Guerrero Vela M, Souza JP, Zhang J. Searching for the optimal rate of medically necessary cesarean delivery. Birth. 2014;4:237-44.

3 - Villar J, Valladares E, Wojdyla D, Zavaleta N, Carroli G, Velazco A, et al; WHO 2005 global survey on maternal and perinatal health research group. Caesarean delivery rates and pregnancy outcomes: the 2005 WHO global survey on maternal and perinatal health in Latin America. Lancet. 2006;367:1819-29.

4 - Timor-Tritsch IE, Monteagudo A. Unforeseen consequences of the increasing rate of cesarean deliveries: early placenta accreta and cesarean scar pregnancy: a review. Am J Obstet Gynecol. 2012;207:14-29.

5 - Brasil. Ministério da Saúde. Secretaria de Vigilância em Saúde. Departamento de Análise de Situação de Saúde. Saúde Brasil 2011: uma análise da situação de saúde e a vigilância da saúde da mulher/Ministério da Saúde, Secretaria de Vigilância em Saúde, Departamento de Análise de Situação de Saúde. Brasília: Editora do Ministério da Saúde, 2012.

6 - SINASC - <http://www2.datasus.gov.br/DATASUS/index.php?area=0205&VObj=http://tabnet.datasus.gov.br/cgi/deftohtm.exe?sinasc/cnv/nv>. Accessed 10 August 2015.

7 - Gibbons L, Belizán JM, Lauer JA, Betrán AP, Merialdi M, Althabe F. The global numbers and costs of additionally needed and unnecessary caesarean sections performed per year: overuse as a barrier to universal coverage. World Health Report (2010) Background Paper, No 30.

8 - Dahlen HG, Tracy S, Tracy M, Bisits A, Brown C, Thornton C. Rates of obstetric intervention among low-risk women giving birth in private and public hospitals in NSW: a population-based descriptive study. BMJ Open. 2012;2:e001723.

9 - Tracy SK, Welsh A, Hall B, Hartz D, Lainchbury A, Bisits A, et al. Caseload midwifery compared to standard or private obstetric care for first time mothers in a public teaching hospital in Australia: a cross sectional study of cost and birth outcomes. BMC Pregnancy Childbirth. 2014;14:46.

10 - Nippita TA, Lee YY, Patterson JA, Ford JB, Morris JM, Nicholl MC, et al. Variation in hospital caesarean section rates and obstetric outcomes among nulliparae at term: a population-based cohort study. BJOG. 2015;122:702-11.

11 -Carmo Leal MD, Pereira AP, Domingues RM, Theme Filha MM, Dias MA,

Nakamura-Pereira M, et al. Obstetric interventions during labor and childbirth in Brazilian low-risk women. Cad Saude Publica. 2014;30 Suppl 1:S1-16.

12 - Zhang J, Troendle J, Reddy UM, Laughon SK, Branch DW, Burkman R, et al.

Consortium on Safe Labor. Contemporary cesarean delivery practice in the United

States. Am J Obstet Gynecol. 2010 Oct;203(4):326.e1-326.e10.

13 - Vasconcellos MTL, Silva PLN, Pereira APE, Schilithz AOC, Souza Junior PRB, Szwarcwald CL. Desenho da amostra *Nascer no Brasil*: Pesquisa Nacional sobre Parto e Nascimento. Cad Saude Publica. 2014;30 Suppl:S49-58.

14 - do Carmo Leal M, da Silva AA, Dias MA, da Gama SG, Rattner D, Moreira ME, et al. Birth in Brazil: national survey into labour and birth. Reprod Health. 2012;9:15.

15 - Victora CG, Huttly SR, Fuchs SC, Olinto MT. The role of conceptual frameworks in epidemiological analysis: a hierarchical approach. Int J Epidemiol. 1997;26:224-7.

16 - Ministério da Saúde. Pré-Natal e Puerpério: atenção qualificada e humanizada. Brasília: Ministério da Saúde; 2006.

17 - Safe prevention of the primary cesarean delivery. Obstetric Care Consensus No. 1. American College of Obstetricians and Gynecologists. Obstet Gynecol 2014;123:693–711.

18 - Bryant AS, Washington S, Kuppermann M, Cheng YW, Caughey AB. Quality and equality in obstetric care: racial and ethnic differences in caesarean section delivery rates. Paediatr Perinat Epidemiol. 2009;23:454-62.

19 - O'Neill SM, Agerbo E, Kenny LC, Henriksen TB, Kearney PM, Greene RA, et al. Cesarean section and rate of subsequent stillbirth, miscarriage, and ectopic pregnancy: a Danish register-based cohort study. PLoS Med. 2014;11:e1001670.

20 - Domingues RMSM, Hartz ZMA, Dias MAB, Leal MC. Avaliação da adequação da assistência pré-natal na rede SUS do Município do Rio de Janeiro, Brasil. Cad Saude Publica. 2012;28:425-37

21 - Coutinho T, Monteiro MFG, Sayd JD, Teixeira MTB, Coutinho CM, Coutinho LM. Monitoring the prenatal care process among users of the Unified Health Care System in a city of the Brazilian Southeast. Rev Bras Ginecol Obstet. 2010;32:563-9.

22 - Carroli G, Rooney C, Villar J. How effective is an- tenatal care in preventing maternal mortality and serious morbidity? An overview of the evidence. Paediatr Perinat Epidemiol. 2001; 15 Suppl 1:1-42

23 - Domingues RM, Dias MA, Nakamura-Pereira M, Torres JA, d’Orsi E, Pereira AP, et al. Process of decision-making regarding the mode of birth in Brazil: from the initial preference of women to the final mode of birth. Cad Saude Publica. 2014;30 Suppl 1:S1-16.

24 - Mealing NM, Roberts CL, Ford JB, Simpson JM, Morris JM. Trends in induction of labour, 1998-2007: a population-based study. Aust N Z J Obstet Gynaecol. 2009;49:599-605.

25 - Recent declines in induction of labor by gestational age. NCHS Data Brief. No. 155. June 2014. <http://www.cdc.gov/nchs/data/databriefs/db155.pdf>. Accessed 10 August 2015.

26 - Wood S, Cooper S, Ross S. Does induction of labour increase the risk of caesarean section? A systematic review and meta-analysis of trials in women with intact membranes. BJOG. 2014;121:674-85.

27 - Mishanina E, Rogozinska E, Thatthi T, Uddin-Khan R, Khan KS, Meads C. Use of labour induction and risk of cesarean delivery: a systematic review and meta-analysis. CMAJ. 2014;186:665-73.

28 - Holmes P, Oppenheimer L, Wen S. The relationship between cervical dilatation at initial presentation in labour and subsequent intervention. Br J Obstet Gynaecol. 2001;108:1120-24.

29 - Janssen PA, Weissinger S. Women’s perception of pre-hospital labour duration and obstetrical outcomes; a prospective cohort study. BMC Pregnancy Childbirth. 2014;14:182.

30 - Neal JL, Lamp JM, Buck JS, Lowe NK, Gillespie SL, Ryan SL. Outcomes of nulliparous women with spontaneous labor onset admitted to hospitals in preactive versus active labor. J Midwifery Womens Health. 2014;59:28-34.

31 - Secretaria de Políticas de Saúde, Ministério da Saúde. Parto, aborto e puerpério: assistência humanizada à mulher. Brasília: Ministério da Saúde; 2001.

32 - World Health Organization, Maternal and Newborn Health/Safe Motherhood Unit. Care in normal birth: a practical guide. Geneva: World Health Organization; 1996.

33 - Singata M, Tranmer J, Gyte GM. Restricting oral fluid and food intake during labour. Cochrane Database Syst Rev. 2010;(1):CD003930.

34 - Lavender T, Hart A, Smyth RM. Effect of partogram use on outcomes for women in spontaneous labour at term. Cochrane Database Syst Rev. 2012;8:CD005461. Update in: Cochrane Database Syst Rev. 2013;7:CD005461.

35 - Eriksen LM, Nohr EA, Kjaergaard H. Mode of delivery after epidural analgesia in a cohort of low-risk nulliparas. Birth. 2011;38:317-26.

36 - Leighton BL, Halpern SH. The effects of epidural analgesia on labor, maternal, and neonatal outcomes: a systematic review. Am J Obstet Gynecol. 2002;186 Suppl 5:S69-77.

37 - Anim-Somuah M, Smyth RM, Jones L. Epidural versus non-epidural or no analgesia in labour. Cochrane Database Syst Rev. 2011;(12):CD000331.

38 - McLachlan HL, Forster DA, Davey MA, Farrell T, Gold L, Biro MA, et al. Effects of continuity of care by a primary midwife (caseload midwifery) on caesarean section rates in women of low obstetric risk: the COSMOS randomised controlled trial. BJOG. 2012;119:1483-92.

39 - Wong N, Browne J, Ferguson S, Taylor J, Davis D. Getting the first birth right: A retrospective study of outcomes for low-risk primiparous women receiving standard care versus midwifery model of care in the same tertiary hospital. Women Birth. 2015;pii:S1871-5192(15)00074-8.

40 - Vogt SE, da Silva KS, Dias MAB. Comparison of childbirth care models in public hospitals, Brazil. Revista de Saude Publica. 2014;48:304-13.

41 - Sandall J, Soltani H, Gates S, Shennan A, Devane D. Midwife-led continuity

models versus other models of care for childbearing women. Cochrane Database Syst

Rev. 2016 Apr 28;4:CD004667. doi: 10.1002/14651858.CD004667.pub5. Review.

42 - National vital statistics report births: final data for 2013. <http://www.cdc.gov/nchs/data/nvsr/nvsr64/nvsr64_01.pdf>. Accessed 10 August 2015.

43 - Delbaere I, Cammu H, Martens E, Tency I, Martens G, Temmerman M. [Limiting the caesarean section rate in low risk pregnancies is key to lowering the trend of increased abdominal deliveries: an observational study.](http://www.ncbi.nlm.nih.gov/pubmed/22230339) BMC Pregnancy Childbirth. 2012;12:3.

44 - Birth in Brazil: national survey into labour and birth.[do Carmo Leal M](http://www.ncbi.nlm.nih.gov/pubmed/?term=do%20Carmo%20Leal%20M%5BAuthor%5D&cauthor=true&cauthor_uid=22913663)1, [da Silva AA](http://www.ncbi.nlm.nih.gov/pubmed/?term=da%20Silva%20AA%5BAuthor%5D&cauthor=true&cauthor_uid=22913663), [Dias MA](http://www.ncbi.nlm.nih.gov/pubmed/?term=Dias%20MA%5BAuthor%5D&cauthor=true&cauthor_uid=22913663), [da Gama SG](http://www.ncbi.nlm.nih.gov/pubmed/?term=da%20Gama%20SG%5BAuthor%5D&cauthor=true&cauthor_uid=22913663), [Rattner D](http://www.ncbi.nlm.nih.gov/pubmed/?term=Rattner%20D%5BAuthor%5D&cauthor=true&cauthor_uid=22913663), [Moreira ME](http://www.ncbi.nlm.nih.gov/pubmed/?term=Moreira%20ME%5BAuthor%5D&cauthor=true&cauthor_uid=22913663), [Filha MM](http://www.ncbi.nlm.nih.gov/pubmed/?term=Filha%20MM%5BAuthor%5D&cauthor=true&cauthor_uid=22913663), [Domingues RM](http://www.ncbi.nlm.nih.gov/pubmed/?term=Domingues%20RM%5BAuthor%5D&cauthor=true&cauthor_uid=22913663), [Pereira AP](http://www.ncbi.nlm.nih.gov/pubmed/?term=Pereira%20AP%5BAuthor%5D&cauthor=true&cauthor_uid=22913663), [Torres JA](http://www.ncbi.nlm.nih.gov/pubmed/?term=Torres%20JA%5BAuthor%5D&cauthor=true&cauthor_uid=22913663), [Bittencourt SD](http://www.ncbi.nlm.nih.gov/pubmed/?term=Bittencourt%20SD%5BAuthor%5D&cauthor=true&cauthor_uid=22913663), [D'orsi E](http://www.ncbi.nlm.nih.gov/pubmed/?term=D'orsi%20E%5BAuthor%5D&cauthor=true&cauthor_uid=22913663), [Cunha AJ](http://www.ncbi.nlm.nih.gov/pubmed/?term=Cunha%20AJ%5BAuthor%5D&cauthor=true&cauthor_uid=22913663), [Leite AJ](http://www.ncbi.nlm.nih.gov/pubmed/?term=Leite%20AJ%5BAuthor%5D&cauthor=true&cauthor_uid=22913663), [Cavalcante RS](http://www.ncbi.nlm.nih.gov/pubmed/?term=Cavalcante%20RS%5BAuthor%5D&cauthor=true&cauthor_uid=22913663), [Lansky S](http://www.ncbi.nlm.nih.gov/pubmed/?term=Lansky%20S%5BAuthor%5D&cauthor=true&cauthor_uid=22913663), [Diniz CS](http://www.ncbi.nlm.nih.gov/pubmed/?term=Diniz%20CS%5BAuthor%5D&cauthor=true&cauthor_uid=22913663), [Szwarcwald CL](http://www.ncbi.nlm.nih.gov/pubmed/?term=Szwarcwald%20CL%5BAuthor%5D&cauthor=true&cauthor_uid=22913663). Reprod Health. 2012 Aug 22;9:15. doi: 10.1186/1742-4755-9-15.

- Idade

- Cor da pele

- Escolaridade

- Trabalho remunerado

- Situação conjugal

- Região

- Adequação do pré-natal

- Condições clinicas e obstetricas potencialmente associadas com emergencias obstetricas antes do nascimento

- Mulheres assistidas na maternidade de referencia

- Procura por serviços de assistencia ao parto antes da admissão hospitalar

- Aconselhamento pré-natal favorável ao parto vaginal

- Decisão pelo tipo de parto ao final da gestação

- Trabalho de parto foi espontâneo ou induzido

- Profissional que assistiu o parto foi o mesmo que fez o pré-natal

- Dilatação Cervical na admissão

- Uso de boas práticas

- Pelo menos uma enfermeira obstetrica durante a assistencia ao trabalho de parto

- Uso de cateter venoso

- Uso de analgesia raquidiana ou epidural

Nível Proximal

Nível Distal

Tipo de trabalho de parto:

Vaginal/forceps

Cesariana durante trabalho de parto

Desfechos

Nível intermediário

Figura 1. Modelo hierárquico de análise das primíparas com gestação única em apresentação cefálica.


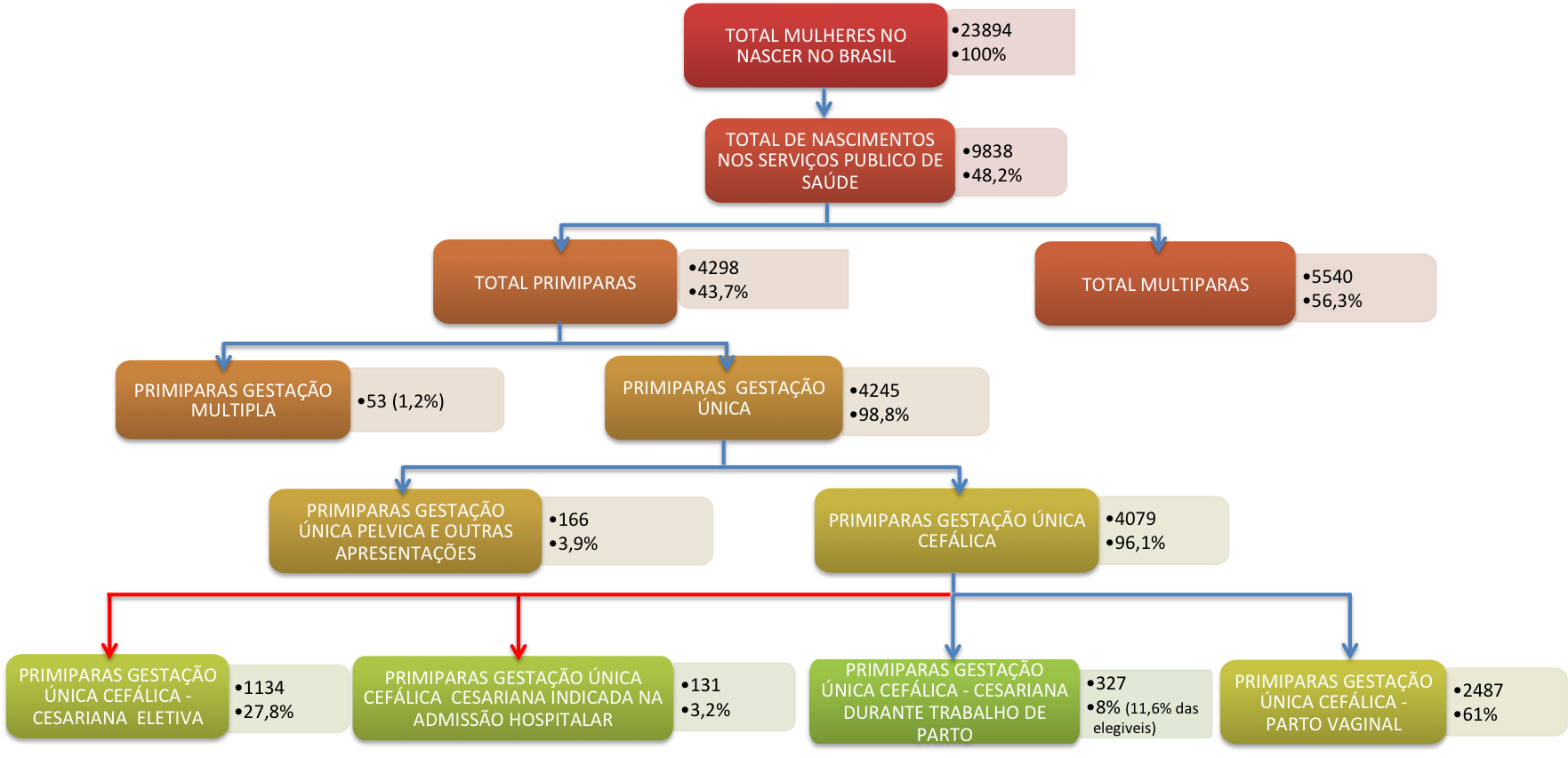


Figura 2. Distribuição das mulheres na amostra do estudo de acordo com o local do nascimento, paridade e outras categorias obstétricas.

Tabela 1. Variáveis Maternas das primíparas assistidas durante o trabalho de parto em hospitais públicos, Brasil, 2011–2012 (N=2487).

|  |  | Tipo de Parto | | | |  |
| --- | --- | --- | --- | --- | --- | --- |
|  |  | Cesariana durante trab. parto | | Parto Vaginal | | valor p*** |
|  |  | N | % | N | % |  |
| Variáveis sociodemograficas | |  |  |  |  |  |
| Idade materna (anos) | 12–19 | 144 | 10.7 | 1198 | 89.3 | 0.372 |
|  | 20–34 | 180 | 12.5 | 1264 | 87.5 |  |
|  | ≥35 | 3 | 10.7 | 25 | 89.3 |  |
| Cor da pele auto-referida | Branca | 69 | 10.4 | 595 | 89.6 | 0.415 |
|  | Preta | 27 | 9.2 | 267 | 90.8 |  |
|  | Parda | 223 | 12.4 | 1569 | 87.6 |  |
| Escolaridade | 0-7 anos | 81 | 11.1 | 650 | 88.9 | 0.753 |
|  | 8-10 anos | 98 | 10.9 | 803 | 89.1 |  |
|  | 11-14 anos | 141 | 12.5 | 984 | 87.5 |  |
|  | 15 ou mais | 6 | 11.5 | 46 | 88.5 |  |
| Trabalho remunerado | Sim | 89 | 12.1 | 649 | 87.9 | 0.728 |
|  | Não | 237 | 11.4 | 1836 | 88.6 |  |
| Situação conjugal | Não vive com parceiro | 83 | 10.5 | 708 | 89.5 | 0.421 |
|  | Vive com parceiro | 243 | 12.0 | 1777 | 88.0 |  |
| Macro região do Brasil | Norte | 50 | 14.0 | 308 | 86.0 | 0.037 |
|  | Nordeste | 147 | 13.9 | 913 | 86.1 |  |
|  | Sul | 29 | 15.3 | 161 | 84.7 |  |
|  | Sudeste | 69 | 6.7 | 960 | 93.3 |  |
|  | Centro-Oeste | 33 | 18.6 | 144 | 81.4 |  |
| Características da gestação, realização de pré-natal e utilização dos serviços de maternidade. | | | |  |  |  |
| Adequação do Pré-Natal | Inadequado | 307 | 11.8 | 2305 | 88.2 | 0.590 |
|  | Adequado | 20 | 9.9 | 182 | 90.1 |  |
| Condições Clinicas ou obstétricas potencialmente associadas com emergências antes do nascimento | Sim | 85 | 22.8 | 288 | 77.2 | < 0.001 |
|  | Não | 242 | 9.9 | 2199 | 90.1 |  |
| Mulher assistida na maternidade de referencia | Sim | 141 | 12.6 | 980 | 87.4 | 0.893 |
|  | Não | 41 | 12.2 | 294 | 87.8 |  |
| Procura por serviços de assistência ao parto antes da admissão hospitalar | Sim | 76 | 13.9 | 470 | 86.1 | 0.134 |
|  | Não | 250 | 11.0 | 2016 | 89.0 |  |
| Aconselhamento pré-natal favorável ao parto vaginal | Sim | 175 | 8.6 | 1854 | 91.4 | <0.001 |
|  | Não | 152 | 19.4 | 633 | 80.6 |  |
| Decisão pelo tipo de parto ao final da gestação | Não | 167 | 17.5 | 790 | 82.5 | <0.001 |
|  | Sim, vaginal | 100 | 5.9 | 1608 | 94.1 |  |
|  | Sim, cesariana | 58 | 45.7 | 69 | 54.3 |  |
| Variáveis relacionadas ao trabalho de parto e parto | |  |  |  |  |  |
| Tipo de trabalho de parto | Espontâneo | 306 | 13.2 | 2018 | 86.8 | <0.001 |
|  | Induzido | 21 | 4.3 | 468 | 95.7 |  |
| Mesmo profissional assistiu o pré-natal e o parto | Sim | 12 | 22.2 | 42 | 77.8 | 0.152 |
|  | Não | 313 | 11.4 | 2442 | 88.6 |  |
| Dilatação Cervical na admissão | ≥4 cm | 171 | 9.7 | 1584 | 90.3 | 0.017 |
|  | <4 cm | 156 | 14.7 | 902 | 85.3 |  |
| Uso de alguma boa prática durante o trabalho de parto | Sim | 207 | 8.7 | 2185 | 91.3 | <0.001 |
|  | Não | 120 | 28.5 | 301 | 71.5 |  |
| Uso de cateter venoso | Sim | 233 | 12.3 | 1656 | 87.7 | 0.507 |
|  | Não | 94 | 10.5 | 800 | 89.5 |  |
| Uso de analgesia raquidiana ou epidural | Sim | 79 | 30.4 | 181 | 69.6 | <0.001 |
|  | Não | 249 | 9.9 | 2276 | 90.1 |  |
| Presença de pelo menos uma enfermeira obstétrica durante a assistência ao trabalho de parto | Sim | 30 | 6.4 | 442 | 93.6 | 0.003 |
|  | Não | 276 | 13.5 | 1774 | 86.5 |  |
| Total |  | 327 | 11.6 | 2487 | 88.4 |  |

*Teste Qui-quadrado Pearson utilizado para análise.

Tabela 2. Regressão logística múltipla das primíparas assistidas no trabalho de parto em hospitais públicos, Brasil, 2011–2012 (N=2487).

|  |  |  | Tipo de Parto | | |
| --- | --- | --- | --- | --- | --- |
|  |  | RR**  Bruto | RR  Ajustado | 95% IC | |
|  |  |  |  | Inferior | Superior |
| Fatores Distais |  |  |  |  |  |
| Macro região do Brasil | Norte | 2,2 | 2.5 | 1.3 | 4.6 |
|  | Nordeste | 2,2 | 2.3 | 1.1 | 4.7 |
|  | Sul | 2,5 | 2.8 | 1.4 | 5.5 |
|  | Sudeste* | 1 | 1 | - | - |
|  | Centro-Oeste | 3,2 | 3.4 | 1.6 | 7.1 |
| Fatores Intermediários |  |  |  |  |  |
| Condições Clinicas ou obstétricas potencialmente associadas com emergências antes do nascimento | Sim | 2,7 | 2.7 | 1.7 | 4.4 |
|  | Não* | 1 | 1 | - | - |
| Aconselhamento pré-natal favorável ao parto vaginal | Sim | 0,4 | 0,5 | 0.3 | 0.6 |
|  | Não* | 1 | 1 | - | - |
| Decisão pelo tipo de parto ao final da gestação | Não | 3,4 | 3.5 | 2.6 | 4.5 |
|  | Sim, vaginal* | 1 | 1 | - | - |
|  | Sim, cesariana | 13,6 | 12.1 | 7.1 | 20.7 |
| Fatores Proximais |  |  |  |  |  |
| Tipo de trabalho de parto | Espontâneo* | 1 | 1 | - | - |
|  | Induzido | 0,3 | 0,2 | 0,1 | 0,4 |
| Dilatação cervical na admissão | ≥4 cm* | 1 | 1 | - | - |
|  | <4 cm | 1,6 | 2.1 | 1.4 | 3.3 |
| Uso de alguma boa prática durante o trabalho de parto | Sim | 0,2 | 0,3 | 0,2 | 0,5 |
|  | Não* | 1 | 1 | - | - |
| Uso de analgesia raquidiana ou epidural | Sim | 4,0 | 3.7 | 1.8 | 7.6 |
|  | Não* | 1 | 1 | - | - |
| Presença de pelo menos uma enfermeira obstétrica durante a assistência ao trabalho de parto | Não | 2,3 | 1.0 | 0.6 | 1.7 |
|  | Sim* | 1 | 1 | - | - |

*Categoria de Referencia **Todos os coeficientes significativos em 0,05.

Tabela 3. Análise bivariada das primíparas assistidas no trabalho de parto em hospitais públicos, Brasil, 2011–2012 (N=2487).

|  |  | Profissional que assistiu ao trabalho de parto | | | |  |
| --- | --- | --- | --- | --- | --- | --- |
|  |  | Médico | | Enf. Obstétrica | | Valor p*** |
|  |  | N | % | N | % |  |
| Uso de alguma boa prática durante o trabalho de parto | Sim | 1709 | 79.4 | 443 | 20.6 | 0.001 |
|  | Não | 342 | 92.2 | 29 | 7.8 |  |
| Beber/alimentar | Sim | 541 | 71.4 | 217 | 28.6 | 0.002 |
|  | Não | 1509 | 85.6 | 254 | 14.4 |  |
| Deambulação | Yes | 961 | 80.2 | 238 | 19.8 | 0.418 |
|  | No | 1089 | 82.3 | 234 | 17.7 |  |
| Uso de método não farmacológico de alívio da dor | Sim | 702 | 74.3 | 243 | 25.7 | 0.006 |
|  | Não | 1349 | 85.5 | 229 | 14.5 |  |
| Presença de acompanhante | Sim | 1101 | 82.8 | 229 | 17.2 | 0.327 |
|  | Não | 790 | 77.7 | 227 | 22.3 |  |
| Monitorar a progressão do trabalho de parto usando um partograma | Sim | 1048 | 74.7 | 355 | 25.3 | <0.001 |
|  | Não | 1003 | 89.6 | 117 | 10.4 |  |
| Uso de analgesia raquidiana ou epidural | Sim | 232 | 92.8 | 18 | 7.2 | 0.002 |
|  | Não | 1818 | 80.1 | 453 | 19.9 |  |
| Total |  | 2050 | 81.3 | 472 | 18.7 |  |
