# Supplementary material for: Factors associated with cesarean delivery during labor in primiparous women assisted in the Brazilian Public Health System: data from a National Survey
Source: Reprod Health. 2016 Oct 17;13(Suppl 3):114. doi: 10.1186/s12978-016-0231-z (PMC5073796; doi:10.1186/s12978-016-0231-z)
Supplement: Additional file 2: — A revisão dos colaboradores para este artigo estão disponíveis no arquivo anexo 2. (PDF 245 kb) [file 12978_2016_231_MOESM2_ESM.pdf]

Referee's comments to the authors– this sheet WILL be seen by the author(s) and published with the article

Title	Factors associated with cesarean delivery during labor in primiparous women who were assisted in the Brazilian Public Health System: Data from a National Survey
Author(s)	Marcos Augusto Dias, Rosa Maria Soares Domingues, Arthur Schilithz, Marcos Nakamura-Pereira and Maria do Carmo Leal
Referee's name	Hannah Dahlen

When assessing the work, please consider the following points, where applicable:

1. **Is the question posed by the authors new and well defined?**
Yes

2. Are the methods appropriate and well described, and are sufficient details provided to replicate the work?
Unclear and unusual

3. Are the data sound and well controlled?
Need for a statistical review

4. Does the manuscript adhere to the relevant standards for reporting and data deposition?
Not stated

5. Are the discussion and conclusions well balanced and adequately supported by the data?
Discussion far too long. Needs to be more focused.

6. Do the title and abstract accurately convey what has been found?
Could be written more tightly

7. Is the writing acceptable?
Needs a good edit.

Please make your report as constructive and detailed as possible in your comments so that authors have the opportunity to overcome any serious deficiencies that you find and please also divide your comments into the following categories:

- Major Compulsory Revisions (which the author must respond to before a decision on publication can be reached)
- Minor Essential Revisions (such as missing labels on figures, or the wrong use of a term, which the author can be trusted to correct)
- Discretionary Revisions (which are recommendations for improvement but which the author can choose to ignore)

Where possible please supply references to substantiate your comments.

When referring to the manuscript please provide specific page and paragraph citations where appropriate.

General comments:
This is an interesting question and of importance to obstetricians, midwives and maternity policy makers. There is a need for substantial editing and cutting down of the discussion to the main significant findings of the study. I would also recommend a statistician review the paper as the approach is an unusual one and quite convoluted.

Major compulsory revisions:
1. I have made comments on the paper to help with editing but it is hard to do a proper edit on a pdf. The paper needs a really good edit
2. The discussion is far to long and needs to be more concise and focus on the significant findings in the paper
3. A statistician needs to review the method

Minor essential revisions:

Referee's comments to the authors– this sheet WILL be seen by the author(s) and published with the article

Title	Factors associated with cesarean delivery during labor in primiparous women who were assisted in the Brazilian Public Health System: Data from a National Survey
Author(s)	Marcos Augusto Dias, Rosa Maria Soares Domingues, Arthur Schilithz, Marcos Nakamura-Pereira and Maria do Carmo Leal
Referee's name	Melissa Amyx

When assessing the work, please consider the following points, where applicable:

1. Is the question posed by the authors new and well defined?
2. Are the methods appropriate and well described, and are sufficient details provided to replicate the work?
3. Are the data sound and well controlled?
4. Does the manuscript adhere to the relevant standards for reporting and data deposition?
5. Are the discussion and conclusions well balanced and adequately supported by the data?
6. Do the title and abstract accurately convey what has been found?
7. Is the writing acceptable?

Please make your report as constructive and detailed as possible in your comments so that authors have the opportunity to overcome any serious deficiencies that you find and please also divide your comments into the following categories:

- Major Compulsory Revisions (which the author must respond to before a decision on publication can be reached)
- Minor Essential Revisions (such as missing labels on figures, or the wrong use of a term, which the author can be trusted to correct)
- Discretionary Revisions (which are recommendations for improvement but which the author can choose to ignore)

Where possible please supply references to substantiate your comments.

When referring to the manuscript please provide specific page and paragraph citations where appropriate.

General comments:

Thank you for the chance to review this relevant article about CS in Brazil. This paper examines factors related to CS during labor in primiparous women in the public health care sector in Brazil, a topic pertinent due to the increasing rates of CS in Brazil and the potential risks of the surgery. The study question is well-defined, although I do not know whether it is new. Overall, study methodology is appropriate and data is sound/well-controlled, with one strength of the study being that the data was collected through a national hospital-based survey of births in Brazil. However, some aspects of the methods can be described better/in more detail (see comments below). The results show that living outside the Southeast Region, having clinical/obstetrical conditions associated with obstetric emergencies, deciding to have a CS late in pregnancy/not making a decision regarding mode of delivery, early admission, and use of analgesia increase the odds of having a CS, while counseling favorable for vaginal delivery, induction of labor, and use of best practices in labor are protective. These results/conclusions contribute to understanding factors related to intrapartum CS and are important for reducing CS rates; however, there appear to be some mistakes in the results presented (see comments below). The discussion/conclusion are well-balanced and adequately supported by the data, but would be strengthened with revision (see comments below). Finally, the title is appropriate and the abstract generally reflects content; the writing is acceptable, but there are minor language issues which would benefit from English language revision if possible. Overall, the manuscript adheres to applicable standards and I believe that with revision, this article will be a valuable contribution to the literature.

(continue on the next sheet)

Continued:

Major compulsory revisions:

1. Several p-values in *Table 3* appear to be incorrect (eating, mobility, use of nonpharmacological pain relief), as well as “same professional provided assistance during ANC and childbirth” in *Table 1*
2. The results (percentages) presented in *Tables 1 and 3* do not appear to be weighted as stated in methods
3. Revision of discussion section: include discussion of strengths (including generalizability) and limitations/bias of the study (one issue to note is the large amount of missing for “linkage to maternity service”); this section is very long and does not flow well
4. What information was used to determine mode of delivery and thereby inclusion/exclusion in this study (elective CS, CS indicated at moment of admission to hospital, CS during labor, or vaginal delivery)?

Minor essential revisions:

Abstract

5. Include N for present analysis; methods do not mention analyses presented in *Table 1 and 3*, no univariate logistic regression/crude ORs presented in paper

Background

6. *Page 4, line 62*: Latest WHO Statement concluded that: “caesarean section rates higher than 10% are not associated with reductions in maternal and newborn mortality rate” http://www.who.int/reproductivehealth/publications/maternal_perinatal_health/cs-statement/en/
7. *Page 4, lines 75-77* (“The excessively interventionist routine….”); *page 5, lines 84-5*: “Among strategies recommended to reduce CD….” provide citations/justification/explanation

Methods

8. How were variables being used collected (which were self-reported by women, from hospital/medical records)?
9. *Page 6, line 110*: “models described in the current literature”; are you referring to models with factors specific to mode of delivery (which you used as a basis for *Figure 1*)? If so, please provide citations.
10. Paid work (*Figure 1*)/remunerated job (*Table 1*) not listed in methods as distal variable (*page 6, lines 112-7*)
11. *Page 6, lines 124-7*: Definition of some variables unclear (“linkage to maternity service”; “search for childbirth care services before hospital admission”; “decision on the type of delivery at the end of pregnancy”)
12. *Page 6, line 130*: “cervical dilation at admission (<4 cm or ≥ then 4 cm)”-specify here that this is considered early admission (as used in other places in manuscript) and refer to as <4cm in written results as opposed to up to 3 cm for consistency (Ex: p*age 9, line 191; page 10, line 215; page 10, line 225*)
13. Outcome/dependent variable defined twice (*page 5, line 105; page 7, line 135-6*)
14. *Page 7, line 138*: Add explanation for statistical analysis presented in *Table 1*; unadjusted ORs not presented
15. *Page 8, line 161-2*: Clarify consent process (was only oral informed consent given, not written?)

Results/tables/figures:

16. Correct inconsistencies and minor errors
    a. *Table 1*: maternal age 12-19, %CS in labor should be 10.7
    b. *Table 2*: Macro region North upper CI should be 4.6
    c. *Figure 2* and *page 8, lines 166-7*: percentages different; 39% repeated
    d. Categorize variables in *Table 1* as distal, intermediate, and proximal factors
    e. Are “linkage to maternity service” (*page 6, line 124; Figure 1, Table 1*) and “delivered in reference maternity” (*page 9, line 183; page 10, line 208*) the same variable?
    f. *Page 10, lines 211-13*: access to diet, freedom of movement, and using non-pharmacological methods for pain relief not presented in *Table 1*
    g. *Page 10 line 226*: OR for decision in late pregnancy for CD different from value in *Table 2*
    h. *Page 10 line 227*: 95% CI for use of analgesia different from values in *Table 2*
17. *Page 8 line 176-page 9 line 197*: This paragraph is very long; would be better presented in a table (alone or incorporated into *Table 1*)
18. What is meant in *Figure 2*: “11,6% of elegibles”?; correct spelling is “eligible”

Discussion

19. *Page 12, lines 263-5*: revise sentence for clarity
20. Remove crossed out text (*page 15, line 355; page 16, lines 361-2*)
21. *Page 16, lines 359-63*: This sentence seems out of place in this paper

Continued:

Discretionary revisions:

22. Results: I would be interested to see results from all models (those discussed in methods *page 7, lines 138-146*)
23. *Page 8, lines 168-70*: wording is a bit confusing, as it makes it sound like you are going to present proportions of surgeries of surgeries, not all births
24. *Page 11, lines 247-52*: these sentences seems out of place

Supplement Editor comments on revised manuscript:
I consider that the manuscript cannot be published in the current version and particularly the Discussion section should be significantly rewritten.
There are many considerations and conclusions that are beyond the results of the study and should be avoided.
Please try to strongly reduce the Discussion section. Please start with a sentence summarizing the major findings of the study.
To redo the Discussion section please try to follow this structure:
* statement of principal findings of the study. Summarise key results with reference to study objectives
* strengths and weaknesses of the study
* strengths and weaknesses in relation to other studies, discussing important differences in results and what your study adds. Whenever possible please discuss your study in the light of relevant systematic reviews and meta-analyses (eg Cochrane reviews)
* meaning of the study: possible explanations and implications for clinicians and policymakers and other researchers; how your study could promote better decisions
* unanswered questions and future research

Minor comments. A description of SUS is needed. International readers are not familiar with the health system in Brazil.
Minor comment. In the abstract, line 26, Instead of "The rate of cesarean section (CD).." It should be cesarean delivery
